# Spatial Segregation Across Travelling Fronts in Individual-Based and Continuum Models for the Growth of Heterogeneous Cell Populations

**DOI:** 10.1007/s11538-025-01452-y

**Published:** 2025-05-19

**Authors:** José A. Carrillo, Tommaso Lorenzi, Fiona R. Macfarlane

**Affiliations:** 1 Mathematical Institute, University of Oxford, Oxford, UK; 2 Department of Mathematical Sciences “G. L. Lagrange”, Politecnico di Torino, Turin, Italy; 3 School of Mathematics and Statistics, University of St Andrews, St Andrews, UK; 4 Applied BioSimulation, Certara (UK), Sheffield, UK

**Keywords:** Phenotypic heterogeneity, Individual-based models, Continuum models, Travelling waves, Spatial segregation

## Abstract

We consider a partial differential equation model for the growth of heterogeneous cell populations subdivided into multiple distinct discrete phenotypes. In this model, cells preferentially move towards regions where they are less compressed, and thus their movement occurs down the gradient of the cellular pressure. The cellular pressure is defined as a weighted sum of the densities (i.e. the volume fractions) of cells with different phenotypes. To translate into mathematical terms the idea that cells with distinct phenotypes have different morphological and mechanical properties, both the cell mobility and the weighted amount the cells contribute to the cellular pressure vary with their phenotype. We formally derive this model as the continuum limit of an on-lattice individual-based model, where cells are represented as single agents undergoing a branching biased random walk corresponding to phenotype-dependent and pressure-regulated cell division, death, and movement. Then, we study travelling wave solutions whereby cells with different phenotypes are spatially segregated across the invading front. Finally, we report on numerical simulations of the two models, demonstrating excellent agreement between them and the travelling wave analysis. The results presented here indicate that inter-cellular variability in mobility can support the maintenance of spatial segregation across invading fronts, whereby cells with a higher mobility drive invasion by occupying regions closer to the front edge.

## Introduction

### Background

Systems of partial differential equations (PDEs) modelling the growth of populations that disperse to avoid crowding (Bertsch et al. 1985; Gurtin and Pipkin 1984) have been applied to describe the spatiotemporal dynamics of multiple types of cells underpinning tissue development, wound healing, and tumour growth (Ambrosi and Preziosi 2002; Bertsch et al. 2012; Bubba et al. 2020; Byrne and Preziosi 2003; Carrillo 2019; Chaplain 2006; Ciarletta et al. 2011; David et al. 2024; Drasdo and Hoehme 2012; Giverso et al. 2022; Gwiazda et al. 2019; Lorenzi et al. 2017; Bertsch et al. 2010; Oelschläger 1989; Roose et al. 2007; Preziosi and Tosin 2009).

Focusing on a one-dimensional spatial scenario, and considering two cell types, a prototypical example of these models is provided by the following PDE system 
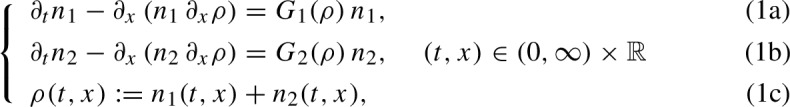
 Here, the functions $n_1(t,x)$ and $n_2(t,x)$ are the densities (i.e. the volume fractions) of cells of types 1 and 2 at position *x* at time *t*, and the function $\rho (t,x)$ defined via (1c) is the total cell density (i.e. the total cell volume fraction). The transport terms on the left-hand sides of the PDEs (1a) and (1b) model the effect of cell movement and capture the tendency of cells to move away from overcrowded regions (i.e. to move down the gradient of the total cell density) (Chaplain 2006). Moreover, the reaction terms on the right-hand sides model the effect of cell division and death, with the functions $G_1(\rho )$ and $G_2(\rho )$ being the net growth rates of the densities of cells of types 1 and 2. These functions depend on the total cell density so as to integrate the effect of density-dependent inhibition of growth (i.e. the cessation of growth at sufficiently high cell density) (Lieberman 1981).

A variation on the model (1) is given by the following PDE system 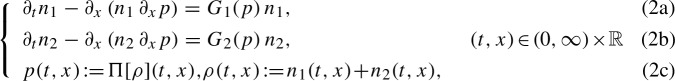 The PDE system (2) can be obtained by replacing the cell density $\rho (t,x)$ in (1) with the cellular pressure *p*(*t*, *x*) and then closing the resulting system for the cell density functions by defining the cell pressure as a function of the total cell density through an appropriate constitutive relation $\Pi [\rho ]$ (Ambrosi and Preziosi 2002; Perthame et al. 2014). This makes it possible to incorporate into the model the effects of pressure-regulated growth – i.e. the fact that cells will stop dividing if the pressure at their current position overcomes a critical value (Byrne and Preziosi 2003; Byrne and Drasdo 2009; Drasdo and Hoehme 2012; Ranft et al. 2010) – and pressure-regulated cell movement – i.e. the movement of cells down the gradient of the cellular pressure towards regions where they are less compressed (Byrne and Chaplain 1997; Byrne and Preziosi 2003).

These models, which provide a population-level description of cell dynamics, can be derived as the continuum limits of underlying individual-based models, which track the dynamics of single cells and are thus able to capture the finer details of single-cell movement, division, and death (Drasdo 2005). To this end, a range of limiting procedures have been developed and employed for transitioning between individual-based models for the growth of cell populations and continuum models formulated as systems of PDEs of the form of (1) and (2) and related forms (Alasio et al. 2022; Carrillo 2019; Chaplain et al. 2020; Dyson et al. 2012; Fozard et al. 2010; Galiano and Selgas 2014; Lorenzi et al. 2020; Penington et al. 2011; Pillay et al. 2017, 2018; Simpson et al. 2011, 2024).

An interesting feature of PDE systems like (1) and (2) is that they can support spatial segregation between different cell types – i.e. the fact that if cells of different types are initially separated (i.e. they occupy distinct regions of the spatial domain at the initial time) then they will remain separated also at later times. Mathematically speaking, this means that the solutions of these PDE systems may exhibit a propagation of segregation property (David et al. 2024). This is an interesting mathematical aspect, the study of which has received increasing attention in recent decades (Bertsch et al. 2015, 2012, 1987a, b, 1985; Burger et al. 2020; Carrillo 2018a, b; Girardin and Hilhorst 2022; Galiano and Selgas 2014; Lorenzi et al. 2017; Bertsch et al. 2010; Falcó et al. 2024). Furthermore, it makes such models an appropriate theoretical framework to investigate the mechanisms underlying cell segregation processes leading to the formation of sharp borders between cells of distinct types or with different phenotypes, which is observed both in normal development and in tumourigenesis (Batlle and Wilkinson 2012).

### Object of Study

In this paper, we consider the following model for the growth of a phenotypically heterogeneous population comprising cells of different types (i.e. with different phenotypes), which are labelled by the index $i=1, \ldots , I$ with $I \ge 2$: 
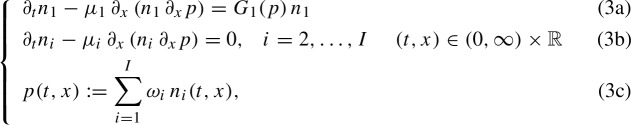


In the model (3), the functions $n_1(t,x), \ldots , n_I(t,x)$ represent the densities (i.e. the volume fractions) of cells with phenotypes $1, \ldots , I$ at position *x* at time *t*, while the function *p*(*t*, *x*) represents the cellular pressure, which is defined as a function of the cell densities through the constitutive relation (3c). The positive parameters $\omega _1, \ldots , \omega _I$ provide a measure of the weighted amounts that cells with phenotypes $1, \ldots , I$ contribute towards the cellular pressure, and the values of these parameters are related to the morphological and mechanical properties of the cells (such as cell size and stiffness), which may vary depending on the cell phenotype (Masaeli et al. 2016). In analogy with model (2), the second terms on the left-hand sides of the PDEs (3a) and (3b) are the rates of change of the densities of cells with phenotypes $1, \ldots , I$ due to pressure-regulated cell movement. The positive parameter $\mu _i$ is the mobility coefficient of cells with phenotype *i* (Ambrosi and Preziosi 2002; Byrne and Drasdo 2009), which in model (2) is implicitly assumed to be the same for all cells (and it is then set to 1), but in fact it can vary due again to differences in morphological and mechanical properties (such as cell elongation and nucleus deformability) between cells with different phenotypes (Kalukula et al. 2022; Lamouille et al. 2014). Moreover, similarly to model (2), the term on the right-hand side of the PDE (3a) is the rate of change of the density of cells with phenotype 1 due to pressure-regulated growth and, therefore, the function $G_1(p)$ is the net growth rate of the density of cells with phenotype 1 when exposed to the cellular pressure *p*. Focusing on the impact of inter-cellular variability in mobility on cell dynamics rather than variability in division and death rates, the right-hand sides of the PDEs (3b) are set to zero. This corresponds to a biological scenario where division and death of cells with phenotypes $2, \ldots , I$ occur on much slower time scales compared to division and death of cells with phenotype 1, and can thus be neglected. For example, in the context of cancer, tumour-derived leader cells undergo epithelial to mesenchymal transition acquiring mesenchymal characteristics including significantly increased motility and significantly reduced proliferation (Chen et al. 2020; Huang et al. 2022; Konen et al. 2017; Vilchez Mercedes et al. 2021; Wang et al. 2023; Zanotelli et al. 2021). Specifically, it has been observed that on short time-scales the proliferation rate of leader cells compared to follower cells is negligible (Konen et al. 2017). Hence, in the framework of model (3), cells with phenotype 1 could be regarded as follower-type cells, while cells with phenotypes $2, \ldots , I$ could be regarded as leader-type cells with heterogeneous morphological and mechanical properties.

In contrast to previous works on related models, which considered two cell phenotypes only (i.e. $i=1,2$) and assumed the values of the weights $\omega _i$ and the values of the mobility coefficients $\mu _i$ to be the same for both phenotypes, in this paper we consider an arbitrary number $I \ge 2$ of cell phenotypes and allow both the values of $\omega _i$ and the values of $\mu _i$ to vary with the phenotype, in order to capture intra-population phenotypic heterogeneity more accurately.

### Outline of the Paper

Building on the modelling framework developed in Chaplain et al. (2020), we first formulate an individual-based model (see Sect. 2) where cells are represented as single agents undergoing phenotype-dependent and pressure-regulated cell division, death, and movement according to a set of rules. These rules result in cells performing a branching biased random walk over the one-dimensional lattice that represents the spatial domain (Hughes 1996; Johnston et al. 2012; Penington et al. 2011). Then, using a limiting procedure analogous to the one that we employed in Bubba et al. (2020); Macfarlane and Chaplain (2020); Macfarlane (2022), we formally derive model (3) as the continuum limit of this individual-based model (see Sect. 2.2 and Appendix A:). After that, generalising the method of proof that developed in Lorenzi et al. (2017), we carry out travelling wave analysis of the model (3) (see Sect. 3) and study, under appropriate assumptions on the function $G_1$ and the mobility coefficients $\mu _1, \ldots , \mu _I$, travelling front solutions wherein cells with phenotypes labelled by different values of the index $i=1, \ldots , I$ are spatially segregated across the front (i.e. they occupy distinct regions of the front). Finally, we report on numerical simulations of the individual-based model and numerical solutions of the PDE model (3), which demonstrate excellent agreement between the two models, thus validating the formal limiting procedure employed to derive the continuum limit of the individual-based model, and confirm the results of travelling wave analysis (see Sect. 4). We conclude with a discussion of the main results obtained and provide a brief overview of possible research perspectives (see Sect. 5).

## Individual-Based and Continuum Models

### Individual-Based Model

Considering a one-dimensional spatial scenario, we let cells be distributed and move along the real line ${\mathbb {R}}$. We introduce the notation ${\mathbb {R}}_{+}:= \{ z \in {\mathbb {R}}: z \ge 0 \}$, ${\mathbb {R}}^*_{+}:= {\mathbb {R}}_{+} \setminus \{0\}$, and ${\mathbb {N}}_0:= {\mathbb {N}} \cup \{ 0 \}$, and then discretise the time variable $t\in {\mathbb {R}}_{+}$ and the space variable $x\in {\mathbb {R}}$ as $t_k=k\tau$ with $k\in {\mathbb {N}}_0$ and $x_j=j\Delta _x$ with $j\in {\mathbb {Z}}$, respectively, where $\tau \in {\mathbb {R}}^*_{+}$ represents the time-step and $\Delta _x \in {\mathbb {R}}^*_{+}$ represents the space-step.

We consider a population comprising cells expressing one amongst $I \ge 2$ distinct discrete phenotypes, each labelled by an index $i = 1,\ldots ,I$, and define the density of cells with phenotype *i* at position $x_j$ at time $t_k$, denoted $n_{i, j}^k$, as

4 $$\begin{aligned} n_{i, j}^k:= \dfrac{N_{i, j}^k}{\Delta _x}, \end{aligned}$$

where $N_{i, j}^k$ is the number of cells with phenotype *i* at position $x_j$ at time $t_k$. Moreover, we define the cellular pressure at position $x_j$ at time $t_k$, denoted $p_j^k$, as a function of $n_{i, j}^k$ through the following constitutive relation:

5 $$\begin{aligned} p_j^k:=\displaystyle {\sum _{i=1}^{I} \omega _i \ n_{i, j}^k}, \quad \text {with}\quad \omega _i\in {\mathbb {R}}_+^*. \end{aligned}$$

**Fig. 1:**
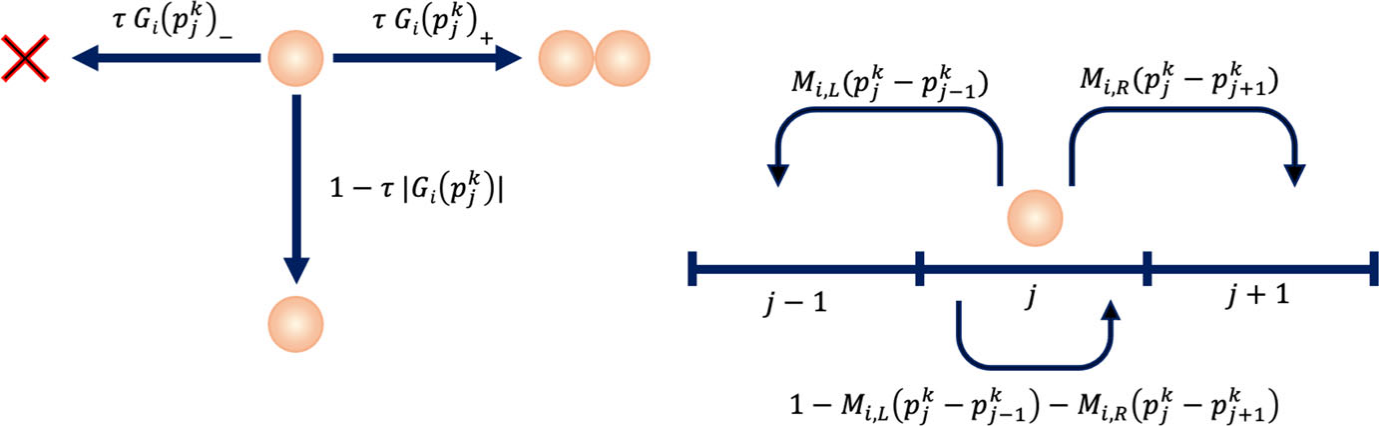
Schematic overview of the mechanisms incorporated in the individual-based model. Between time-steps *k* and $k+1$, each cell with phenotype $i = 1,\ldots ,I$ at spatial position $x_j$ may: divide with probability $\tau \, G_i(p_j^k)_+$, die with probability $\tau \, G_i(p_j^k)_-$, and remain quiescent with probability $1-\tau \, \left( G_i(p_j^k)_+ + G_i(p_j^k)_- \right) = 1 - \tau \, |G_i(p_j^k)|$ (left panel); move to spatial positions $x_{j-1}$ or $x_{j+1}$ with probabilities $M_{i,L}(p_j^k-p_{j-1}^k)$ or $M_{i,R}(p_j^k-p_{j+1}^k)$, respectively, or remain stationary with probability $1-M_{i,L}(p_j^k-p_{j-1}^k)-M_{i,R}(p_j^k-p_{j+1}^k)$ (right panel)

As mentioned in Sect. 1, the constitutive relation (5) translates into mathematical terms the idea that cells with each phenotype *i* may contribute a different weighted amount, which is represented by the parameter $\omega _i$, to the cellular pressure. The value of the parameter $\omega _i$ is related to the morphological and mechanical properties of the cells; for instance, higher values of $\omega _i$ may correspond to larger cell size and/or larger cell stiffness (Masaeli et al. 2016). The dynamics of the cells are governed by the rules summarised in Fig. 1 and detailed in the following subsections.

#### Modelling Cell Division and Death

We model pressure-regulated cell division and death as a branching process along the spatial dimension, whereby cells divide and die with probabilities that depend on both their phenotype and the pressure they experience, as illustrated by the schematic in the left panel of Fig. 1. If cell division occurs, a dividing cell is instantly replaced by two identical progeny cells that inherit the spatial position and phenotype of the parent cell. Conversely, a cell undergoing cell death is instantly removed from the population.

We introduce the function $G_i(p_j^k)$, which represents the net growth rate of the density of cells with phenotype $i=1,\ldots ,I$ at spatial position $x_j$ at time $t_k$, and use this function to define the probability of cell division and death in the individual-based model. Specifically, between time-steps *k* and $k+1$ we let a cell with phenotype *i* at position $x_j$ divide with probability

$$\begin{aligned} \tau G_i(p_j^k)_+, \; \text { where } \; (\cdot )_+=\max (0,\cdot ), \end{aligned}$$

die with probability

$$\begin{aligned} \tau G_i(p_j^k)_-, \; \text { where } \; (\cdot )_-=-\min (0,\cdot ), \end{aligned}$$

and remain quiescent with probability

$$\begin{aligned} 1-\tau \left( G_i(p_j^k)_+ + G_i(p_j^k)_- \right) = 1 - \tau |G_i(p_j^k)|. \end{aligned}$$

By choosing the time-step $\tau$ sufficiently small, we ensure that all the quantities above are between 0 and 1.

In order to take into account the fact that cells will stop dividing if the pressure at their current position exceeds a critical value, known as the homeostatic pressure (Basan et al. 2009), which is modelled by the parameter $\overline{p} \in {\mathbb {R}}_+^*$, and considering a scenario in which cells with phenotypes labelled by different values of the index *i* may undergo cell division and death over different time scales, we make the following assumptions:

6 $$\begin{aligned} G_i(p):= \alpha _i \, G(p), \quad G(0)< \infty , \quad G(\overline{p})=0, \quad \frac{\textrm{d}G}{\textrm{d}p}<0 \, \forall p\in {\mathbb {R}}_{+}. \end{aligned}$$

Here the parameter $\alpha _i \in {\mathbb {R}}_{+}$ is linked to the time scale over which cells with phenotype *i* undergo cell division and death. In particular, focusing on the case where division and death of cells with phenotypes $i=2, \ldots , I$ can be neglected, since they occur on much slower time scales compared to division and death of cells with phenotype $i=1$, we also assume

7 $$\begin{aligned} \alpha _1 > 0 \;\; \text { and } \;\; \alpha _i = 0 \; \text { for } \; i=2, \ldots , I. \end{aligned}$$

#### Modelling Cell Movement

We model directional cell movement in response to pressure differentials as a biased random walk, whereby the movement probabilities depend on the difference between the cellular pressure at the position occupied by the cell and the cellular pressure at neighbouring positions, as illustrated by the schematic in the right panel of Fig. 1. In particular, we assume that cells move down the gradient of the pressure so as to reach regions where they are less compressed. Moreover, in order to capture the fact that the phenotype of the cells determines their sensitivity to the pressure gradient, and thus their mobility, we introduce the parameters $\gamma _i\in {\mathbb {R}}_+^*$ to model the sensitivity to the pressure gradient of cells with phenotypes $i=1,\ldots ,I$.

In the individual-based model, between time-steps *k* and $k+1$ we let a cell with phenotype *i* at position $x_j$ move to position $x_{j-1}$ (i.e. move left) with probability

$$\begin{aligned} {M}_{i,L}(p_j^k-p_{j-1}^k), \end{aligned}$$

move to position $x_{j+1}$ (i.e. move right) with probability

$$\begin{aligned} {M}_{i,R}(p_j^k-p_{j+1}^k), \end{aligned}$$

and remain stationary with probability

$$\begin{aligned} 1-\left( {M}_{i,L}(p_j^k-p_{j-1}^k)+{M}_{i,R}(p_j^k-p_{j+1}^k)\right) . \end{aligned}$$

Specifically, recalling that, as described in Sect. 2.1.1, the parameter $\overline{p}$ represents the homeostatic pressure, we use the following definitions

8 $$\begin{aligned} \begin{array}{l} \quad {M}_{i,L}(p_j^k-p_{j-1}^k)=\displaystyle {\gamma _i \frac{\left( p_j^k-p_{j-1}^k\right) _+}{2\overline{p}}},\\ \quad {M}_{i,R}(p_j^k-p_{j+1}^k)=\displaystyle {\gamma _i \frac{\left( p_j^k-p_{j+1}^k\right) _+}{2\overline{p}}}, \end{array}\quad \text {where} \quad (\cdot )_+=\max (0,\cdot ), \end{aligned}$$

and choose the model parameters and functions such that $0 \le {M}_{i,L}(p_j^k-p_{j-1}^k)+{M}_{i,R}(p_j^k-p_{j+1}^k)<1$ for all *i*, *j*, and *k*.

Without loss of generality, considering a scenario where cells with phenotypes labelled by smaller values of the index *i* have a lower sensitivity to the pressure gradient, and thus a lower mobility, we assume

9 $$\begin{aligned} 0<\gamma _1<\gamma _2<\ldots<\gamma _{I-1}<\gamma _I. \end{aligned}$$

##### Remark 1

Taken together, assumptions (6)-(7) and (9) correspond to the situation in which, due to a trade-off between cell proliferative and migratory abilities, fast-dividing cells with phenotype $i=1$ display the lowest mobility (Chen et al. 2020; Huang et al. 2022; Konen et al. 2017; Vilchez Mercedes et al. 2021; Wang et al. 2023; Zanotelli et al. 2021).

### Corresponding Continuum Model

As detailed in Appendix A:, from the branching biased random walk underlying the individual-based model presented in the previous section, one formally derives, as the corresponding continuum limit, a PDE system for the functions $n_i(t,x)$, each modelling the density (i.e. the volume fraction) of cells with phenotype $i=1, \ldots , I$, at position $x \in {\mathbb {R}}$ at time $t\in {\mathbb {R}}_+$. This is done through an extension of the limiting procedure that we employed in Bubba et al. (2020); Macfarlane and Chaplain (2020); Macfarlane (2022) – see also Simpson et al. (2007); Johnston et al. (2012); Simpson et al. (2011, 2024) for related strategies.

In summary, one writes down a balance equation for the density of cells with phenotype *i* at spatial position $x_j$ at time $t_{k+1}$, which depends on cell densities at time $t_{k}$ at position $x_j$ and neighbouring positions $x_{j-1}$ and $x_{j+1}$, as a result of cell movement and cell division and death. Specifically, one has

10 $$\begin{aligned} n_{i,\ j}^{k+1}= &  n_{i,\ j}^k\left\{ \left( 1+\tau G_i(p_j^k)\right) \left[ 1-\frac{\gamma _i \left( p_j^k-p_{j+1}^k\right) _+}{2\overline{p}}-\frac{\gamma _i \left( p_j^k-p_{j-1}^k\right) _+}{2\overline{p}}\right] \right\} \nonumber \\ &  +n_{i,\ j+1}^k\left\{ \left( 1+\tau G_i(p_{j+1}^k)\right) \left[ \frac{\gamma _i \left( p_{j+1}^k-p_{j}^k\right) _+}{2\overline{p}}\right] \right\} \nonumber \\ &  +n_{i,\ j-1}^k\left\{ \left( 1+\tau G_i(p_{j-1}^k)\right) \left[ \frac{\gamma _i \left( p_{j-1}^k-p_{j}^k\right) _+}{2\overline{p}}\right] \right\} . \end{aligned}$$

From the balance equation (10), employing a formal limiting procedure that includes letting $\Delta _x\rightarrow 0$ and $\tau \rightarrow 0$ in such a way that

11 $$\begin{aligned} \frac{\gamma _i \Delta _x^2}{2\tau \overline{p}}\rightarrow \mu _i, \quad \text {where} \quad \mu _i \in {\mathbb {R}}^*_+,\ i=1, \ldots , I, \end{aligned}$$

under the constitutive relation (5) one formally obtains the PDE system

12 $$\begin{aligned} {\left\{ \begin{array}{ll} \begin{array}{l} \displaystyle {\partial _t n_i - \mu _i \, \partial _x \left( n_i \, \partial _x p \right) = G_i(p) \, n_i, \quad i=1, \ldots , I}, \\ \\ \displaystyle {p(t,x):= \sum _{i=1}^I \omega _i \, n_i(t,x),} \end{array} \quad (t,x) \in (0,\infty ) \times {\mathbb {R}}, \end{array}\right. } \end{aligned}$$

subject to the following assumptions on the mobility coefficients, $\mu _i$,

13 $$\begin{aligned} 0< \mu _1< \mu _2< \ldots< \mu _{I-1} < \mu _I. \end{aligned}$$

Assumptions (13) descend from assumptions (9) when conditions (11) hold. Under the additional assumptions (6)-(7) the PDE system (12) then reduces to the PDE system (3).

## Travelling Wave Analysis

In this section, under assumptions (6)-(7) and (13), we carry out travelling wave analysis of the continuum model (3). Substituting the travelling wave ansatz

$$\begin{aligned} n_i(t,x) = n_i(z), \quad z:= x-c \, t, \quad c \in {\mathbb {R}}^*_+, \end{aligned}$$

where *c* is the travelling wave speed, into the model (3) yields 
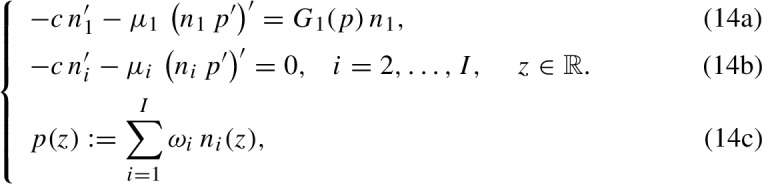
 We seek travelling wave solutions that satisfy the following conditions

15 $$\begin{aligned} n_i(z) {\left\{ \begin{array}{ll} > 0, \;\; \text {for } z \in (z_{i-1}, z_i) \\ =0, \;\; \text {for } z \not \in (z_{i-1}, z_i), \end{array}\right. } \quad i=1, \ldots , I, \end{aligned}$$

where

16 $$\begin{aligned} -\infty =: z_0< z_1:= 0< z_2< \ldots< z_{I-1}< z_I < \infty , \end{aligned}$$

along with the asymptotic (i.e. boundary) condition

17 $$\begin{aligned} p(-\infty ) = \overline{p}. \end{aligned}$$

Note that, under conditions (15)-(16), conservation of mass ensures that

18 $$\begin{aligned} \int _{z_{i-1}}^{z_i} n_i(z) \, \textrm{d}z = M_i, \quad M_i \in {\mathbb {R}}^*_+, \quad i=2, \ldots , I, \end{aligned}$$

where the parameter $M_i$ represents the number (i.e. the total volume fraction) of cells with phenotype $i=2, \ldots , I$ in the population.

### Remark 2

**Fig. 2:**
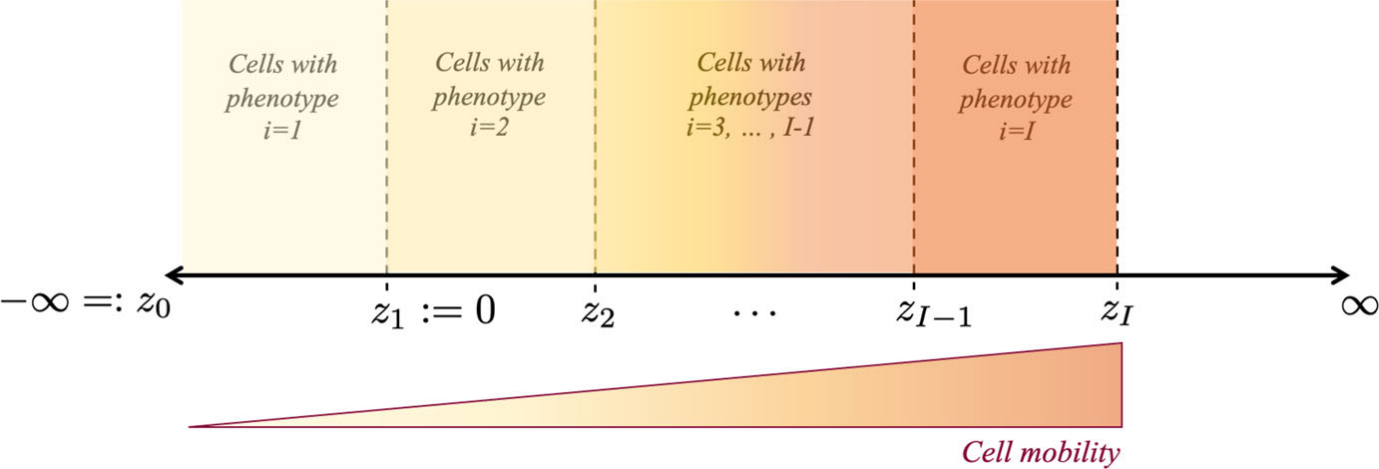
Schematic overview of spatial segregation across travelling waves. Schematic of how, under assumptions (6)-(7) and (13), cells with phenotypes labelled by different values of the index *i* are spatially segregated across invading fronts, which are represented by travelling wave solutions of the continuum model (3), i.e. solutions of the system of differential equations (14), subject to conditions (15)-(16)

Conditions (15)-(16) correspond to a scenario in which cells with phenotypes labelled by different values of the index *i* are spatially segregated across invading fronts, which are represented by travelling wave solutions of the continuum model (3), i.e. solutions of the system of differential equations (14). More precisely, also in the light of assumptions (6)-(7) and (13), cells with phenotype $i=1$ (i.e. fast-dividing cells with the lowest mobility) make up the bulk of the population in the rear of the wave, while cells with phenotypes labelled by increasing values of $i>1$, which display a higher mobility, are found in the regions closer to the invading edge (cf. the schematic in Fig. 2).

The properties of such travelling wave solutions are established by Theorem 1.

### Theorem 1

Let assumptions (6)-(7) and (13) hold. For any $M_2, \ldots , M_I \in {\mathbb {R}}^*_+$ there exist $z_2, \ldots , z_{I} \in {\mathbb {R}}^*_+$ and $c \in {\mathbb {R}}^*_+$ such that the system of differential equations (14) subject to conditions (15)-(17) admits a solution wherein each component $n_i(z)$ is positive, continuous, and decreasing on $(z_{i-1}, z_i)$ for $i=1, \ldots , I$, and the components $n_{2}(z), \ldots , n_{I}(z)$ satisfy conditions (18) as well. Moreover, the function *p*(*z*) defined via the constitutive relation (14c) is positive, continuous, and decreasing on $(-\infty ,z_I)$, with

19 $$\begin{aligned} p(0) = \sqrt{2 \, c \, \sum _{j=2}^I \dfrac{\omega _j}{\mu _j} \, M_j}, \end{aligned}$$

and it has a kink at the points $0, z_2, \ldots , z_{I-2}, z_{I-1}$ with

20 $$\begin{aligned}&-\mu _{2} \, p'(0^+) = -\mu _{1} \, p'(0^-) = c, \;\; \nonumber \\ &-\mu _{i+1} \, p'(z_i^+) = -\mu _{i} p'(z_i^-) = c, \; i=2,\ldots , I-1. \end{aligned}$$

### Proof

We prove Theorem 1 in 8 steps. In summary, building on the shooting method that we employed in Lorenzi et al. (2017), first we prove, for $c \in {\mathbb {R}}^*_+$ given, that: (i) The cell pressure *p*(*z*) and the cell density $n_i(z)$ are positive, continuous, and monotonically decreasing on the intervals $(z_{i-1},z_{i})$ for all $i=1,\ldots ,I$. Then *p*(*z*) is not only monotonically decreasing on each interval but as a whole in ${\mathbb {R}}$, with possibly jumps at the points $z_i$ for $i=1,\ldots ,I-1$. This along with the non-negativity of *p*(*z*) ensures that $0 \le p(z) \le \overline{p}$ for all $z \in {\mathbb {R}}$ (*Step 1*). (ii) The cell pressure is continuous also in $z_i$ for all $i=1,\ldots ,I$ (*Step 2*). (iii) The derivative of the cell pressure satisfies conditions (20) (*Steps 3-4*). Then, still for $c \in {\mathbb {R}}^*_+$ given, imposing conditions (18), we find the values attained by the cell pressure *p*(*z*) at the points $z_2, \ldots , z_{I-1}$, and we prove that condition (19) holds (*Steps 5-6*). Next, still for $c \in {\mathbb {R}}^*_+$ given, we identify the points $z_2, \ldots , z_{I}$ (*Step 7*). Finally, we prove that there exists a unique pair (*c*, *p*) that satisfies the travelling wave problem (*Step 8*).

*Preliminary observations.* Throughout the proof we will be exploiting the fact that the system of differential equations (14) subject to conditions (15)-(16) can be rewritten as 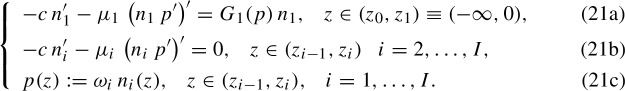 Moreover, multiplying both sides of the differential equation (21a) by $\omega _{1}$ and both sides of the differential equation (21b) by $\omega _{i}$, using the relation between *p* and $n_1$ and *p* and $n_i$ given by (21c) along with the fact that $G_1(p):= \alpha _1 \, G(p)$ where $\alpha _1 > 0$ (cf. assumptions (6)-(7)), we obtain the following set of differential equations for *p*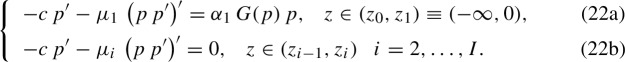 Furthermore, we also notice that, introducing the notation

23 $$\begin{aligned} \mu (z):= \sum _{i=1}^I \mu _i \, \mathbbm {1}_{(z_{i-1},z_{i})}(z), \end{aligned}$$

where $\mathbbm {1}_{(\cdot )}(z)$ is the indicator function of the set $(\cdot )$, and using the fact that, under conditions (15)-(16), both $p(z) = 0$ and $p'(z) = 0$ for all $z >z_I$, the set of differential equations (22b) can be rewritten in a more compact form as

24 $$\begin{aligned} -c \, p'(z) - \left( \mu (z) \, p(z) \, p'(z) \right) ' = 0, \quad z \in (z_1,\infty ) \equiv (0,\infty ). \end{aligned}$$

*Step 1.* For $c \in {\mathbb {R}}^*_+$ given, under assumptions (6) on the function *G*(*p*), the differential equation (22a) subject to the condition (17) admits solutions which are positive, continuous and, by the maximum principle, monotonically decreasing on $(z_0,z_1) \equiv (-\infty ,0)$. Moreover, still for $c \in {\mathbb {R}}^*_+$ given, integrating the differential equation (24) between $z \in (z_{i-1},z_i)$ with $i=2, \ldots , I$ and $\infty$, and using the fact that both $p(z) \rightarrow 0$ and $p'(z) \rightarrow 0$ as $z \rightarrow \infty$, yields

25 $$\begin{aligned} p'(z) = -\dfrac{c}{\mu _i} \quad z \in (z_{i-1},z_i), \quad i=2, \ldots , I, \end{aligned}$$

from which we see that the differential equation (24) admits solutions which are positive, continuous, and monotonically decreasing on $(z_{i-1},z_{i})$ for all $i=2,\ldots ,I$. In particular, note that integrating the differential equation (25) with $i=I$ between a generic point $z \in (z_{I-1},z_I)$ and $z_I$ and imposing the condition $p(z_I)=0$ (cf. the conditions (15)-(16)) we obtain

26 $$\begin{aligned} p(z) = \dfrac{c}{\mu _I} \, (z_I - z), \quad z \in (z_{I-1},z_I). \end{aligned}$$

In summary, *p*(*z*) is positive, continuous, and monotonically decreasing on $(z_{i-1}, z_i)$ for all $i=1, \ldots , I$. This along with the relations (21c) allow us to conclude that also $n_i(z)$ is positive, continuous, and monotonically decreasing on $(z_{i-1}, z_i)$ for all $i=1, \ldots , I$.

*Step 2.* For $c \in {\mathbb {R}}^*_+$ given, under assumptions (6)-(7), introducing the additional notation

27 $$\begin{aligned} \alpha (z):= \sum _{i=1}^I \alpha _i \, \mathbbm {1}_{(z_{i-1},z_{i})}(z), \end{aligned}$$

and using the definition (23) of $\mu (z)$ along with the fact that, under conditions (15)-(16), both $p(z) = 0$ and $p'(z) = 0$ for all $z >z_I$, we further rewrite the set of differential equations (22) in a more compact form as

28 $$\begin{aligned} -c \, p'(z) - \left( \mu (z) \, p(z) \, p'(z) \right) ' = \alpha (z) \, G(p) \, p(z), \quad z \in {\mathbb {R}}. \end{aligned}$$

Multiplying by *p* both sides of the differential equation (28) and rearranging terms we find

29 $$\begin{aligned} \mu \, p \left( p' \right) ^2 = \alpha \, G(p) \, p^2 + \left( p \, \mu \, p \, p' \right) ' + c \, p \, p', \quad z \in {\mathbb {R}}. \end{aligned}$$

Since the function *p*(*z*) is continuous on $(z_{j-1},z_j)$ for all $j=1, \ldots , I-1$, there exists $z_{j}^* \in (z_{j-1},z_{j})$ such that $p'(z_{j}^*) > -\infty$ for any $j=1, \ldots , I-1$. Hence, integrating both sides of (29) between $z^*_j$ and $z_{j+1}$ and estimating the right-hand side from above, by using the fact that the functions *p*, $\mu$, $\alpha$, and *G*(*p*) are non-negative and bounded on $(z_{j-1}, z_{j+1})$ while the function $p'$ is non-positive on $(z_{j-1}, z_{j+1})$, we obtain

$$\begin{aligned} \int _{z^*_j}^{z_{j+1}} \mu \, p \, \left( p' \right) ^2 \textrm{d}z < \infty , \quad \quad j=1, \ldots , I-1. \end{aligned}$$

The above estimates ensure that $p' \in L^2_{loc}\left( (z^*_j, z_{j+1})\right)$ for $j=1, \ldots , I-1$. This along with the fact that $p \in L^{\infty }({\mathbb {R}})$ allow us to conclude that *p* is also continuous in each $z_i$ for $i=1, \ldots , I-1$, i.e.

30 $$\begin{aligned} p(z_{i}^+) = p(z_{i}^-) = p(z_i), \quad i=1,\ldots ,I-1. \end{aligned}$$

*Step 3.* For $c \in {\mathbb {R}}^*_+$ given, integrating the differential equation (28) between a generic point $z \in (z_{j-1},z_{j+1})$ and $z_{j+1}$ for $j=1,\ldots ,I-1$, and using the fact that *p*(*z*) is continuous and $\mu (z_{j+1}^-)=\mu _{j+1}$ (cf. the definition (23) of $\mu (z)$), yields

31 $$\begin{aligned} c \, p(z) + \mu (z) \, p(z) \, p'(z)= &  \int _{z}^{z_{j+1}} \alpha \, G(p) \, p \, \textrm{d}\zeta \nonumber \\ &  + \, c \, p(z_{j+1}) + \mu _{j+1} \, p(z_{j+1}) \, p'(z_{j+1}^-). \end{aligned}$$

Letting $z \rightarrow z_j^-$ in (31) and using the fact that *p*(*z*) is continuous and $\mu (z_{j}^-)=\mu _{j}$ (cf. the definition (23) of $\mu (z)$) gives

32 $$\begin{aligned} c \, p(z_j) + \mu _j \, p(z_j) \, p'(z_j^-)= &  \int _{z_j}^{z_{j+1}} \alpha \, G(p) \, p \, \textrm{d}z \nonumber \\ &  + \, c \, p(z_{j+1}) + \mu _{j+1} \, p(z_{j+1}) \, p'(z_{j+1}^-). \end{aligned}$$

Similarly, letting $z \rightarrow z_j^+$ in (31) and using the fact that *p*(*z*) is continuous and $\mu (z_{j}^+)=\mu _{j+1}$ (cf. the definition (23) of $\mu (z)$) gives

33 $$\begin{aligned} c \, p(z_j) + \mu _{j+1} \, p(z_j) \, p'(z_j^+)= &  \int _{z_j}^{z_{j+1}} \alpha \, G(p) \, p \, \textrm{d}z \nonumber \\ &  + \, c \, p(z_{j+1}) + \mu _{j+1} \, p(z_{j+1}) \, p'(z_{j+1}^-). \end{aligned}$$

Combining (32) and (33) we obtain

34 $$\begin{aligned} \mu _{i+1} \, p'(z_i^+) = \mu _{i} \, p'(z_i^-), \quad i=1,\ldots , I-1. \end{aligned}$$

*Step 4.* For $c \in {\mathbb {R}}^*_+$ given, combining (25) with $i=I$ (i.e. the fact that $c = - \mu _I \, p'(z_{I-1}^+)$) and the condition (34) with $i=I-1$ (i.e. the fact that $\mu _{I-1} \, p'(z_{I-1}^-)=\mu _I \, p'(z_{I-1}^+)$) we obtain

35 $$\begin{aligned} - \mu _{I-1} \, p'(z_{I-1}^-) = - \mu _I \, p'(z_{I-1}^+) = c. \end{aligned}$$

Moreover, integrating the differential equation (24) between $z_{i}$ and $z_{i+1}$ for $i=1,\ldots ,I-2$ and using the fact that *p*(*z*) is continuous and $\mu (z_{i+1}^-)=\mu (z_{i}^+)=\mu _{i+1}$ (cf. the definition (23) of $\mu (z)$) yields

$$\begin{aligned} &  - c \left( p(z_{i+1}) - p(z_{i})\right) - \mu _{i+1} \left( p(z_{i+1}) \, p'(z_{i+1}^-) - p(z_{i}) \, p'(z_{i}^+) \right) = 0, \\ &  \quad i=1,\ldots ,I-2, \end{aligned}$$

from which, rearranging terms, we find

36 $$\begin{aligned} p(z_{i}) \, \left( c + \mu _{i+1} p'(z_{i}^+)\right) -p(z_{i+1}) \, \left( c + \mu _{i+1} \, p'(z_{i+1}^-) \right) = 0, \quad i=1,\ldots ,I-2.\nonumber \\ \end{aligned}$$

Choosing $i=I-2$ in (36) gives

$$\begin{aligned} p(z_{I-2}) \, \left( c + \mu _{I-1} p'(z_{I-2}^+)\right) -p(z_{I-1}) \, \left( c + \mu _{I-1} \, p'(z_{I-1}^-) \right) = 0 \end{aligned}$$

and then substituting (35) into the above equation yields

$$\begin{aligned} p(z_{I-2}) \, \left( c + \mu _{I-1} \, p'(z_{I-2}^+)\right) = 0 \quad \Longrightarrow \quad - \mu _{I-1} \, p'(z_{I-2}^+) = c, \end{aligned}$$

from which, using condition (34) with $i=I-2$ (i.e. the fact that $\mu _{I-1} \, p'(z_{I-2}^+) = \mu _{I-2} \, p'(z_{I-2}^-)$), we obtain

37 $$\begin{aligned} -\mu _{I-2} \, p'(z_{I-2}^-) = -\mu _{I-1} \, p'(z_{I-2}^+) = c. \end{aligned}$$

Proceeding in a similar way for $i=I-3$ and so on and so forth for $i=I-4, \ldots , 1$ we also find

38 $$\begin{aligned} - \mu _{i} \, p'(z_{i}^-) = - \mu _{i+1} \, p'(z_{i}^+) = c, \quad i=1,\ldots ,I-3. \end{aligned}$$

Taken together, the results given by (35), (37), and (38) allow us to conclude that

39 $$\begin{aligned} -\mu _{i+1} \, p'(z_i^+) = -\mu _{i} \, p'(z_i^-) = c, \quad i=1,\ldots , I-1, \end{aligned}$$

and thus conditions (20) hold.

*Step 5.* For $c \in {\mathbb {R}}^*_+$ given, the expression (26) for *p* on $(z_{I-1},z_I)$ along with the constitutive relation (21c) with $i=I$ yield

40 $$\begin{aligned} n_{I}(z) = \dfrac{c}{\omega _I \, \mu _I} (z_I - z), \quad z \in (z_{I-1},z_I). \end{aligned}$$

Substituting (40) into (18) with $i=I$, and taking the positive root so as to ensure that the condition $z_{I}-z_{I-1}>0$ (i.e. $z_{I} > z_{I-1}$) holds, gives

41 $$\begin{aligned} \dfrac{c}{\omega _I \, \mu _I} \int _{z_{I-1}}^{z_I} (z_I - z) \, \textrm{d}z = M_I \quad \Longrightarrow \quad z_I = z_{I-1} + \sqrt{\dfrac{2 \, \omega _I \, \mu _I}{c} \, M_I}. \end{aligned}$$

Finally, substituting the expression (41) for $z_I$ into (26) and evaluating the resulting expression for *p*(*z*) in $z=z_{I-1}$ yields

$$\begin{aligned} p(z_{I-1}^+) = \sqrt{\dfrac{2 \, c \, \omega _I}{\mu _I} \, M_I}, \end{aligned}$$

from which, exploiting the fact that *p*(*z*) is continuous, we obtain

42 $$\begin{aligned} p(z_{I-1}) = \sqrt{\dfrac{2 \, c \, \omega _I}{\mu _I} \, M_I}. \end{aligned}$$

*Step 6.* For $c \in {\mathbb {R}}^*_+$ given, integrating the differential equation (25) between a generic point $z \in (z_{i-1},z_i)$ and $z_i$ for $i=2,\ldots ,I-1$, we find

43 $$\begin{aligned} p(z) = p(z_i) + \dfrac{c}{\mu _i} (z_i - z), \quad z \in (z_{i-1}, z_i), \quad i=2,\ldots ,I-1. \end{aligned}$$

Choosing $i=I-1$ in (43) gives

44 $$\begin{aligned} p(z) = p(z_{I-1}) + \dfrac{c}{\mu _{I-1}} (z_{I-1} - z), \quad z \in (z_{I-2}, z_{I-1}). \end{aligned}$$

Integrating (44) between $z_{I-2}$ and $z_{I-1}$, using the fact that $p(z) = \omega _{I-1} n_{I-1}(z)$ for $z \in (z_{I-2}, z_{I-1})$ (cf. the constitutive relation (21c) with $i=I-1$), and imposing the condition (18) with $i=I-1$ gives

$$\begin{aligned} \int _{z_{I-2}}^{z_{I-1}} \left( p(z_{I-1}) + \dfrac{c}{\mu _{I-1}} (z_{I-1} - z) \right) \, \textrm{d}z = \omega _{I-1} \, M_{I-1}, \end{aligned}$$

from which, computing the integral, solving the resulting quadratic equation for $z_{I-1}-z_{I-2}$, and taking the positive root so as to ensure that the condition $z_{I-1}-z_{I-2}>0$ (i.e. $z_{I-1} > z_{I-2}$) holds, we find

45 $$\begin{aligned} z_{I-1} - z_{I-2} = \sqrt{\left( \dfrac{\mu _{I-1}}{c}\right) ^2 (p(z_{I-1}))^2 + 2 \, \dfrac{\mu _{I-1}}{c} \, \omega _{I-1} \, M_{I-1}} - \dfrac{\mu _{I-1}}{c} \, p(z_{I-1}).\nonumber \\ \end{aligned}$$

Moreover, evaluating (44) in $z_{I-2}$ and substituting (45) into the resulting equation yields

$$\begin{aligned} p(z_{I-2})= &  \dfrac{c}{\mu _{I-1}} \, \sqrt{\left( \dfrac{\mu _{I-1}}{c}\right) ^2 (p(z_{I-1}))^2 + 2 \, \dfrac{\mu _{I-1}}{c} \, \omega _{I-1} \, M_{I-1}}\\= &  \sqrt{(p(z_{I-1}))^2 + \dfrac{2 \, c \, \omega _{I-1}}{\mu _{I-1}} \, \, M_{I-1}}. \end{aligned}$$

Finally, substituting the expression (42) for $p(z_{I-1})$ into the above equation we obtain

46 $$\begin{aligned} p(z_{I-2}) = \sqrt{\dfrac{2 \, c \, \omega _I}{\mu _I} \, M_I + \dfrac{2 \, c \, \omega _{I-1}}{\mu _{I-1}} \, \, M_{I-1}}. \end{aligned}$$

Proceeding in a similar way for $i=I-2$ and so on and so forth for $i=I-3, \ldots , 2$ we also find

47 $$\begin{aligned} p(z_i) = \sqrt{2 \, c \, \sum _{j=i+1}^I \dfrac{\omega _j}{\mu _j} \, M_j}, \quad i=2,\ldots ,I-2. \end{aligned}$$

Taken together, the results given by (42), (46), and (47) allow us to conclude that

48 $$\begin{aligned} p(z_i) = \sqrt{2 \, c \, \sum _{j=i+1}^I \dfrac{\omega _j}{\mu _j} \, M_j}, \quad i=1,\ldots ,I-1. \end{aligned}$$

Choosing $i=1$ in (48) and recalling that $z_1:=0$ (cf. conditions (16)), we obtain

49 $$\begin{aligned} p(0) = \sqrt{2 \, c \, \sum _{j=2}^I \dfrac{\omega _j}{\mu _j} \, M_j}, \end{aligned}$$

which implies that condition (19) holds.

*Step 7.* Evaluating (43) in $z_{i-1}$ and solving for $z_{i}$ yields

50 $$\begin{aligned} z_i = z_{i-1} + \dfrac{\mu _i}{c} \left( p(z_{i-1}) - p(z_i)\right) , \quad i=2, \ldots , I-1. \end{aligned}$$

Choosing $i=2$ in (50) and recalling that $z_1:=0$ (cf. conditions (16)) gives

$$\begin{aligned} z_2 = \dfrac{\mu _2}{c} \left( p(0) - p(z_2)\right) \end{aligned}$$

and then, substituting into the above equation the expression (49) for *p*(0) and the expression for $p(z_2)$ obtained by choosing $i=2$ in (48), we find

51 $$\begin{aligned} z_2 = \dfrac{\mu _2}{c} \left( \sqrt{2 \, c \, \sum _{j=2}^I \dfrac{\omega _j}{\mu _j} \, M_j} - \sqrt{2 \, c \, \sum _{j=3}^I \dfrac{\omega _j}{\mu _j} \, M_j}\ \right) . \end{aligned}$$

Moreover, proceeding in a similar way for $i=3$ and so on and so forth for $i=4, \ldots , I-1$ we also obtain

52 $$\begin{aligned} z_i = z_{i-1} + \dfrac{\mu _i}{c} \left( \sqrt{2 \, c \, \sum _{j=i}^I \dfrac{\omega _j}{\mu _j} \, M_j} - \sqrt{2 \, c \, \sum _{j=i+1}^I \dfrac{\omega _j}{\mu _j} \, M_j}\ \right) , \quad i=3, \ldots , I-1.\nonumber \\ \end{aligned}$$

Finally, from (41) we find

53 $$\begin{aligned} z_I = z_{I-1} + \sqrt{\dfrac{2 \, \omega _I \, \mu _I}{c} \, M_I}, \end{aligned}$$

with $z_{I-1}$ obtained from (52) by choosing $i=I-1$.

*Step 8.* Complementing the differential equation (22a) with the condition (17) and the condition (39) for $i=1$, we obtain the following problem 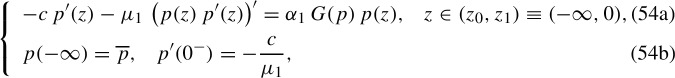 from which, proceeding as similarly done in Lorenzi et al. (2017), it is possible to prove (see Appendix B:) that $c \mapsto p(0^-)$ is monotonically decreasing. On the other hand, the expression (49) for *p*(0) implies that $c \mapsto p(0^+)$ is monotonically increasing. These facts along with the condition $p(0^-)=p(0^+)$, which follows from the continuity of *p*(*z*), allow us to conclude that there exists a unique pair (*c*, *p*) that satisfies the travelling wave problem. $\square$

### Remark 3

The fact that, as established by Theorem 1, the cell pressure *p*(*z*) defined via the constitutive relation (14c) is continuous throughout the support of the travelling wave gives the following interface conditions for the cell densities

55 $$\begin{aligned} n_{i+1}(z_{i}^+) = \dfrac{\omega _{i}}{\omega _{i+1}} n_{i}(z_{i}^-), \quad i=1, \ldots , I-1. \end{aligned}$$

These conditions imply that if $\omega _{i}<\omega _{i+1}$ then $n_{i+1}(z_{i}^+)<n_{i}(z_{i}^-)$, whereas if $\omega _{i} \ge \omega _{i+1}$ then $n_{i+1}(z_{i}^+) \ge n_{i}(z_{i}^-)$.

### Remark 4

The conditions (20) imply that

$$\begin{aligned} -p'(z_i^+) = - \dfrac{\mu _i}{\mu _{i+1}} \, p'(z_i^-), \quad i=1, \ldots , I-1 \end{aligned}$$

and, therefore, since $p'(z_i^+)<0$ and $p'(z_i^-)<0$ for all $i=1, \ldots , I-1$, we have

$$\begin{aligned} |p'(z_i^+)| = \dfrac{\mu _i}{\mu _{i+1}} \, |p'(z_i^-)|, \quad i=1, \ldots , I-1. \end{aligned}$$

Under assumptions (13), which imply that $\dfrac{\mu _i}{\mu _{i+1}}<1$ for all $i=1, \ldots , I-1$, the above relations allow us to conclude that

$$\begin{aligned} |p'(z_i^+)| < |p'(z_i^-)|, \quad i=1, \ldots , I-1. \end{aligned}$$

## Numerical Simulations

In this section, we present results of numerical simulations of the individual-based model introduced in Sect. 2 and the corresponding continuum model defined by the PDE system (3), and compare them to the results of travelling wave analysis obtained in Sect. 3. In particular, we investigate the cases where there are either three or four different cellular phenotypes (i.e. $I=3$ or $I=4$).

### Set-Up of Numerical Simulations

We carry out numerical simulations over the spatial domain [0, *L*], with $L=150$. In order to ensure that assumptions (6) are satisfied, we use the following definition of the function *G*(*p*), both when $I=3$ and when $I=4$,

56 $$\begin{aligned} G(p):=\arctan \left( \dfrac{1}{10}\left( 1-\frac{p}{\overline{p}}\right) \right) . \end{aligned}$$

Furthermore, we choose values of the parameters $\alpha _i$ and $\mu _i$ satisfying assumptions (7) and (13). Specifically, in the case where $I=3$ we use the following baseline parameter values

57 $$\begin{aligned} &  \alpha _1=10,\quad \alpha _2=\alpha _3=0,\nonumber \\ &  \mu _1=10^{-4},\quad \mu _2=2\times 10^{-4},\quad \mu _3=3\times 10^{-4},\nonumber \\ &  \omega _1=1,\quad \omega _2=2,\quad \omega _3=3, \end{aligned}$$

and in the case where $I=4$ we complement the parameter choice given by (57) with

58 $$\begin{aligned} \alpha _4=0, \quad \mu _4=4\times 10^{-4}, \quad \omega _4=4. \end{aligned}$$

In both cases, we explore also deviations of the weights $\omega _i$ from these values in the numerical simulations.

Moreover, in line with the travelling wave analysis carried out in Sect. 3, we investigate propagation of segregation properties by letting cells with different phenotypes be spatially segregated at $t=0$. Specifically, both for the individual-based model and for the continuum model, when $I=3$ we define the initial cell densities as

59 $$\begin{aligned} n_1(0,x)= &  A_1 \exp \left[ -B \, x^2\right] \mathbbm {1}_{[0,10)}(x),\nonumber \\ n_2(0,x)= &  A_2 \exp \left[ -B \, (x-10)^2\right] \mathbbm {1}_{[10,20)}(x),\nonumber \\ n_3(0,x)= &  A_3 \exp \left[ -B \, (x-20)^2\right] \mathbbm {1}_{[20,L)}(x), \end{aligned}$$

while when $I=4$ we use the following definitions for the initial cell densities

60 $$\begin{aligned} n_1(0,x)= &  A_1 \exp \left[ -B \, x^2\right] \mathbbm {1}_{[0,10)}(x),\nonumber \\ n_2(0,x)= &  A_2 \exp \left[ -B \, (x-10)^2\right] \mathbbm {1}_{[10,20)}(x),\nonumber \\ n_3(0,x)= &  A_3 \exp \left[ -B \, (x-20)^2\right] \mathbbm {1}_{[20,30)}(x),\nonumber \\ n_4(0,x)= &  A_4 \exp \left[ -B \, (x-30)^2\right] \mathbbm {1}_{[30,L)}(x). \end{aligned}$$

In (59) and (60), the function $\mathbbm {1}_{(\cdot )}(x)$ is the indicator function of the set $(\cdot )$, $B=6\times 10^{-2}$, and the parameters $A_i$ are positive real numbers. We choose the homeostatic pressure to be

$$\begin{aligned} \overline{p} = 4\times 10^4 \end{aligned}$$

and, given this value of $\overline{p}$ and the values selected for the weights $\omega _i$ in (3c) and (5), we then choose the values of the parameters $A_i$ in (59) and (60) such that the initial cell pressure satisfies $p(0,x)\le \overline{p}$ for all $x \in [0,L]$ and is consistent between numerical simulations.

Finally, to carry out numerical simulations of the individual-based model, we choose the space-step $\Delta _x=0.1$, the time-step $\tau =1\times 10^{-4}$, and we define

61 $$\begin{aligned} \gamma _i:= \frac{2\tau \overline{p}}{\Delta _x^2} \mu _i \end{aligned}$$

so as to ensure that conditions (11) underlying the formal derivation of the continuum model are met. Note that, since we choose values of the parameters $\mu _i$ satisfying assumptions (13), defining the values of the parameter $\gamma _i$ according to (61) ensures that assumptions (9) are satisfied as well.

### Computational Implementation of the Individual-Based Model and Numerical Scheme for the Continuum Model

All simulations are performed in Matlab. For the individual-based model, at each time-step, every individual cell can undergo: (i) movement, according to the probabilities defined in Sect. 2.1.2; (ii) division and death, according to the probabilities defined in Sect. 2.1.1. For each of these processes a random number is drawn from the standard uniform distribution on the interval (0, 1) using the built-in Matlab function rand. If this random number is smaller than the probability of the event occurring then the process is successful. To impose zero-flux boundary conditions, any attempted move outside the spatial domain is aborted. To numerically solve the PDE system (3) subject to zero-flux boundary conditions we use a finite volume scheme modified from our previous works (Bubba et al. 2020; Lorenzi et al. 2023). The full details of this scheme are provided in Appendix C.

**Fig. 3:**
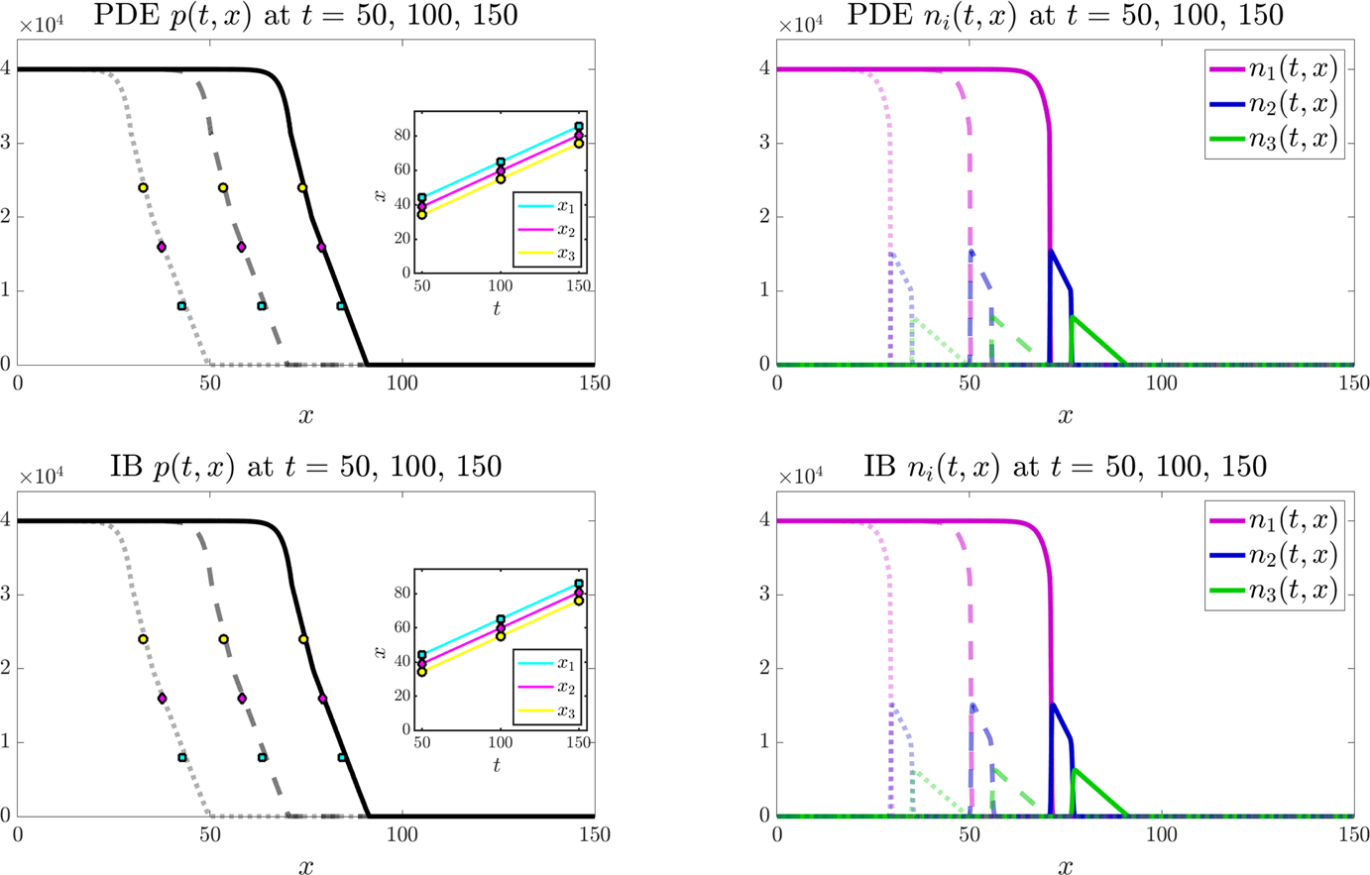
Main results under the baseline parameter setting for $I=3$. Comparing numerical solutions of the continuum model (top panels) with the averaged results of 10 simulations of the individual-based model (bottom panels), when $I=3$ and the values of the parameters $\alpha _i$, $\mu _i$, and $\omega _i$ are set according to (57). Plots display the cell pressure *p*(*t*, *x*) (left panels) and the cell densities $n_i(t,x)$ (right panels) at three successive time instants – i.e. $t=50$ (dotted lines), $t=100$ (dashed lines), and $t=150$ (solid lines). The insets of the left panels display the plots of $x_1(t)$ (cyan), $x_2(t)$ (magenta), and $x_3(t)$ (yellow) defined via (62). The coloured markers in the plot of *p*(*t*, *x*) highlight the values of $p(t,x_1(t))$ (cyan), $p(t,x_2(t))$ (magenta), and $p(t,x_3(t))$ (yellow) at $t=50$, $t=100$, and $t=150$. The numerically estimated wave speeds are $c_{\text {PDEn}}= 0.42$ and $c_{\text {IBn}}=0.42$, and the analytically predicted wave speed is $c_{\text {a}}=0.42$ (Color figure online)

### Main Results of Numerical Simulations

**Fig. 4:**
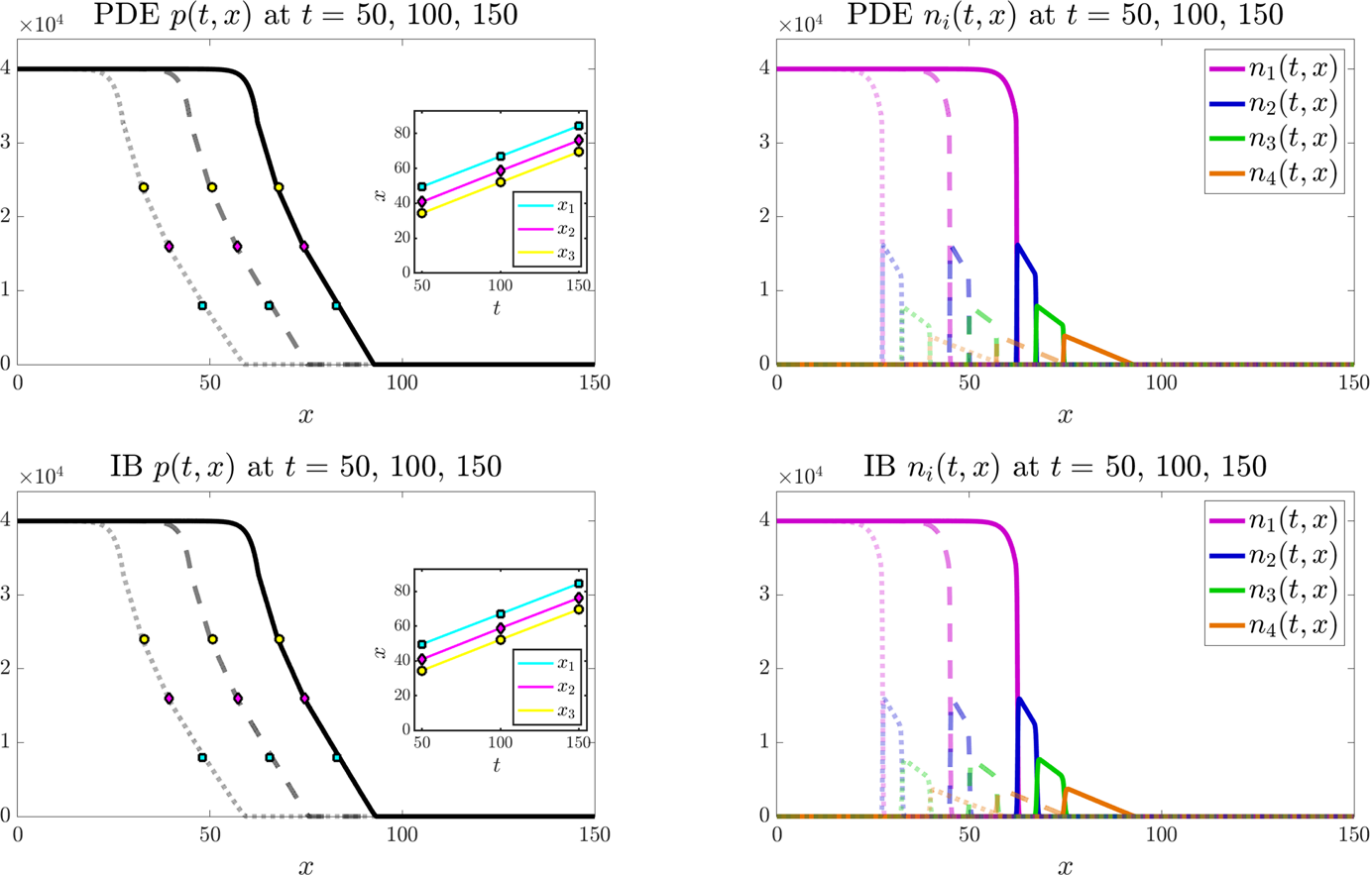
Main results under the baseline parameter setting for $I=4$. Comparing numerical solutions of the continuum model (top panels) with the averaged results of 10 simulations of the individual-based model (bottom panels), when $I=4$ and the values of the parameters $\alpha _i$, $\mu _i$, and $\omega _i$ are set according to (57)-(58). Plots display the cell pressure *p*(*t*, *x*) (left panels) and the cell densities $n_i(t,x)$ (right panels) at three successive time instants – i.e. $t=50$ (dotted lines), $t=100$ (dashed lines), and $t=150$ (solid lines). The insets of the left panels display the plots of $x_1(t)$ (cyan), $x_2(t)$ (magenta), and $x_3(t)$ (yellow) defined via (62). The coloured markers in the plot of *p*(*t*, *x*) highlight the values of $p(t,x_1(t))$ (cyan), $p(t,x_2(t))$ (magenta), and $p(t,x_3(t))$ (yellow) at $t=50$, $t=100$, and $t=150$. The numerically estimated wave speeds are $c_{\text {PDEn}}= 0.35$ and $c_{\text {IBn}}=0.35$, and the analytically predicted wave speed is $c_{\text {a}}=0.35$ (Color figure online)

Figures 3 and 4 display the results of numerical simulations of the individual-based model (bottom panels) and the corresponding continuum model defined by the PDE system (3) (top panels) for $I=3$ and $I=4$, respectively, under the baseline parameter settings (57) and (57)-(58), respectively. Moreover, Figs. 6 and 7 display the results of numerical simulations of the continuum model for $I=3$ and $I=4$, respectively, when the values of the parameters $\alpha _i$ and $\mu _i$ are set according to (57) and (57)-(58), respectively, while different combinations of the weights $\omega _i$ are considered.

#### Agreement between the individual-based and the continuum models

**Fig. 5:**
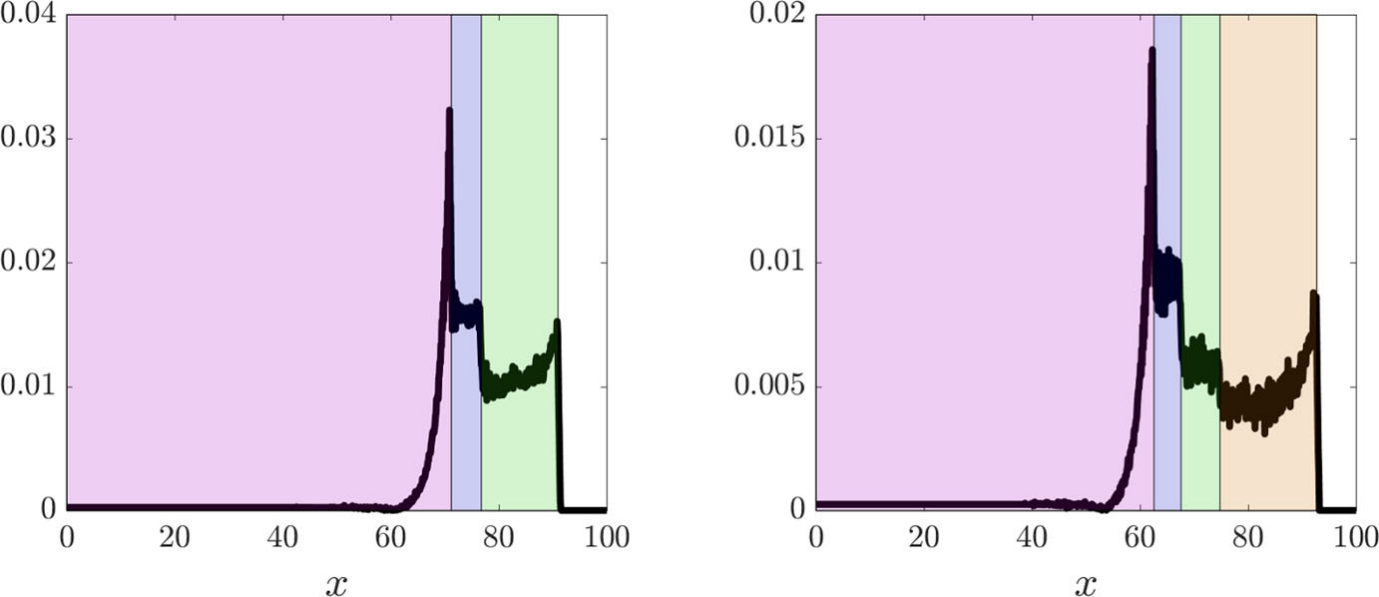
Quantitative comparison between the individual-based and the continuum models. Plot of the quantity $\dfrac{|p_{\text{ P }DE}(t,x) - p_{\text{ I }B}(t,x)|}{\overline{p}}$ at $t=150$, where $p_{\text{ P }DE}$ is the cell pressure computed from numerical solutions of the continuum model displayed in Fig. 3 (left panel) and Fig. 4 (right panel), while $p_{\text{ I }B}$ is the cell pressure computed from the averaged results of 10 simulations of the individual-based model displayed in the same figures. The supports of the cell densities $n_i$ for $i=1,\ldots , I$ with $I=3$ (left panel) or $I=4$ (right panel) are highlighted in the same colours as those of the curves of the cell densities displayed in Figs. 3 and 4 (Color figure online)

**Fig. 6:**
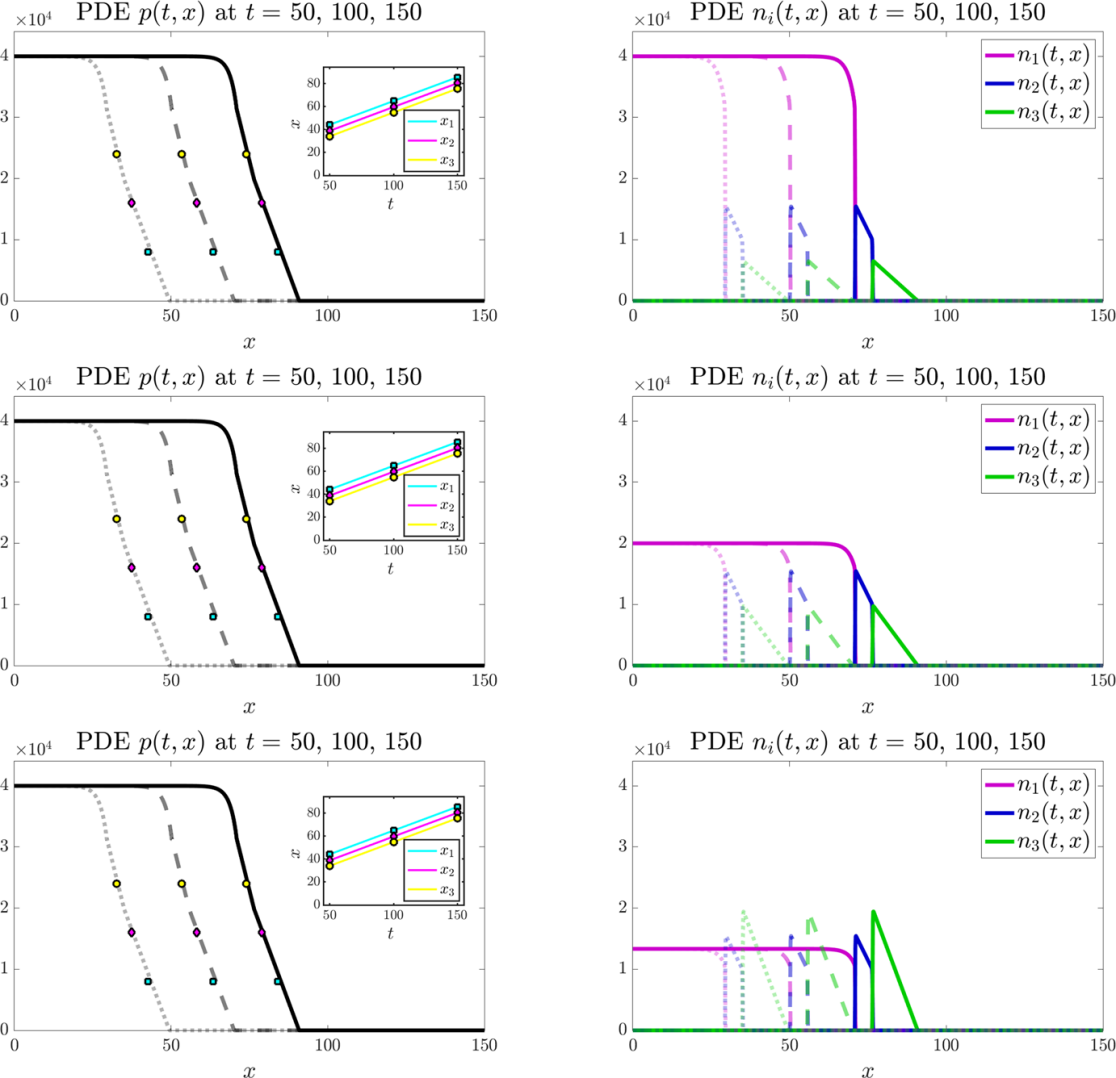
Main results under different values of the parameters $\omega _i$ for $I=3$. Numerical solutions of the continuum model when $I=3$ and the values of the parameters $\alpha _i$ and $\mu _i$ are set according to (57), while the values of the parameters $\omega _i$ are: ${\omega _1=1,\ \omega _2=2,\ \omega _3=3}$ (top panels); ${\omega _1=2,\ \omega _2=2,\ \omega _3=2}$ (central panels); and ${\omega _1=3,\ \omega _2=2,\ \omega _3=1}$ (bottom panels). Plots display the cell pressure *p*(*t*, *x*) (left panels) and the cell densities $n_i(t,x)$ (right panels) at three successive time instants – i.e. $t=50$ (dotted lines), $t=100$ (dashed lines), and $t=150$ (solid lines). The insets of the left panels display the plots of $x_1(t)$ (cyan), $x_2(t)$ (magenta), and $x_3(t)$ (yellow) defined via (62). The coloured markers in the plot of *p*(*t*, *x*) highlight the values of $p(t,x_1(t))$ (cyan), $p(t,x_2(t))$ (magenta), and $p(t,x_3(t))$ (yellow) at $t=50$, $t=100$, and $t=150$. For all cases we report on in this figure, the numerically estimated wave speed is $c_{\text {PDEn}}= 0.42$, while the analytically predicted wave speed is $c_{\text {a}}=0.42$ (Color figure online)

**Fig. 7:**
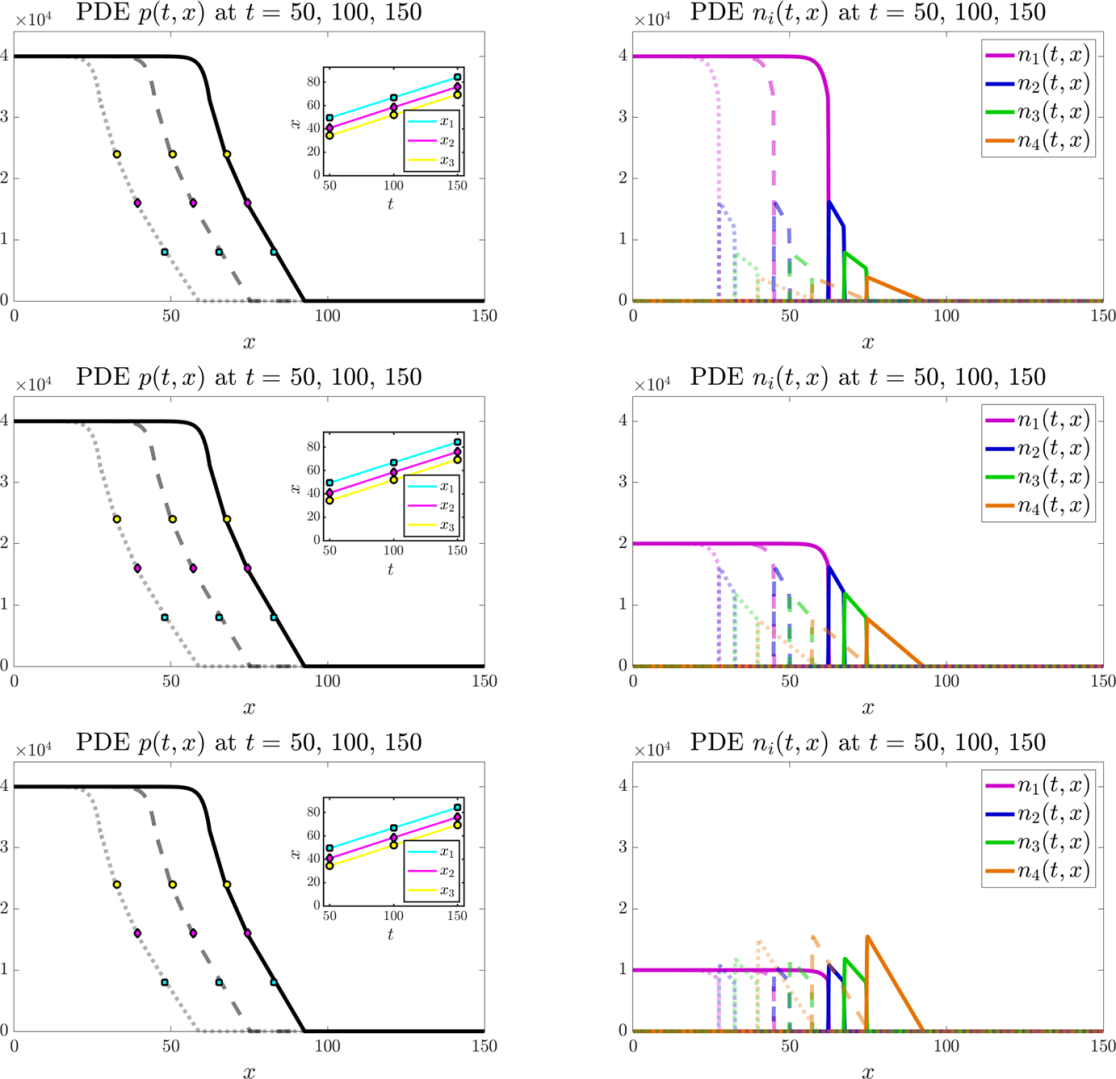
Main results under different values of the parameters $\omega _i$ for $I=4$. Numerical solutions of the continuum model when $I=4$ and the values of the parameters $\alpha _i$ and $\mu _i$ are set according to (57)-(58), while the values of the parameters $\omega _i$ are: ${\omega _1=1,\ \omega _2=2,\ \omega _3=3,\ \omega _4=4}$ (top panels); ${\omega _1=2,\ \omega _2=2,\ \omega _3=2,\ \omega _4=2}$ (central panels); and ${\omega _1=4,\ \omega _2=3,\ \omega _3=2,\ \omega _4=1}$ (bottom panels). Plots display the cell pressure *p*(*t*, *x*) (left panels) and the cell densities $n_i(t,x)$ (right panels) at three successive time instants – i.e. $t=50$ (dotted lines), $t=100$ (dashed lines), and $t=150$ (solid lines). The insets of the left panels display the plots of $x_1(t)$ (cyan), $x_2(t)$ (magenta), and $x_3(t)$ (yellow) defined via (62). The coloured markers in the plot of *p*(*t*, *x*) highlight the values of $p(t,x_1(t))$ (cyan), $p(t,x_2(t))$ (magenta), and $p(t,x_3(t))$ (yellow) at $t=50$, $t=100$, and $t=150$. For all cases we report on in this figure, the numerically estimated wave speed is $c_{\text {PDEn}}= 0.35$, while the analytically predicted wave speed is $c_{\text {a}}=0.35$ (Color figure online)

The results summarised by the plots in Figs. 3 and 4 demonstrate that overall there is excellent agreement between numerical simulations of the individual-based model and numerical solutions of the corresponding continuum model, as expected since conditions (11) are met. Moreover, although, for the sake of clarity, only the results of numerical simulations of the continuum model are displayed in Figs. 6 and 7, we verified that also for the parameter settings corresponding to these figures the results of numerical simulations of the individual-based model agree with those of the continuum model (results not shown). We stress that possible quantitative discrepancies between the two models emerge in the proximity of the interfaces between regions occupied by cells with different phenotypes (see Fig. 5). This is because in these areas there is a stronger interplay between demographic stochasticity and sharp transitions in cell densities, which causes a reduction in the quality of the continuum approximations that are employed in the formal derivation of the PDE model from the underlying individual-based model. We also remark that the quantitative agreement between the two models may deteriorate in scenarios where a limited number of cells is considered, since in these scenarios the impact of demographic stochasticity is amplified.

#### Propagation of travelling fronts

The numerical results in Figs. 3-4 and Figs. 6-7 show the propagation of travelling fronts wherein, as expected from Theorem 1, the cell densities $n_i$ have disjoint supports, which means that cells with different phenotypes occupy distinct regions across the front (cf. right panels). In particular, as captured by assumptions (6)-(7) and (13), cells with phenotypes labelled by larger values of the index *i*, which display a higher migratory ability, occupy regions closer to the edge of the front, while fast-dividing cells with the lowest mobility (i.e. cells with phenotype $i=1$) make up the bulk of the population in the rear of the front. Moreover, also in agreement with Theorem 1, the cell pressure *p* is continuous throughout the wave, whereas its first spatial derivative exhibits jump discontinuities at the interfaces between the regions occupied by cells with different phenotypes (cf. left panels). Specifically, after an initial transient during which the travelling front is formed, the numerical values of the first spatial derivative of the cell pressure *p* are such that the interface conditions (20) are satisfied.

In order to confirm the propagation of travelling waves, we also track the dynamics of the points $x_1(t)$, $x_2(t)$, and $x_3(t)$ such that

62 $$\begin{aligned} p(t,x_1(t))=0.2 \, \overline{p}, \quad p(t,x_2(t))=0.4 \, \overline{p}, \quad p(t,x_3(t))=0.6 \, \overline{p}, \end{aligned}$$

and verify that, after an initial transient during which the travelling wave is formed, the functions $x_1(t)$, $x_2(t)$, and $x_3(t)$ behave like straight lines with approximately the same constant slope (cf. insets of the left panels). We numerically estimate the wave speeds for the individual-based model and for the continuum model, denoted $c_{\text {IBn}}$ and $c_{\text {PDEn}}$, respectively, by measuring the slope of $x_1(t)$ after the transient. We then compare the numerically estimated wave speeds with the analytically predicted one, denoted $c_{\text {a}}$, which is obtained through (19), i.e. substituting into the following formula

$$\begin{aligned} c_{\text {a}} = \dfrac{\left( p(0)\right) ^2}{\displaystyle {2 \sum _{j=2}^I \dfrac{\omega _j}{\mu _j} \, M_j}} \end{aligned}$$

the values of $M_2, \ldots , M_I$ and the value of *p*(0) estimated from numerical solutions of the continuum model. In particular, we approximate $M_j$ with the numerical value of the integral of the cell density $n_j$ over the spatial domain for $j=2,\ldots ,I$, which remains constant over time, while *p*(0) is approximated as the numerical value of the cell pressure *p* at the right endpoint of the support of the cell density $n_{1}$ at a time *t* large enough that the travelling wave is established. We find that there is good agreement between the values of $c_{\text {IBn}}$, $c_{\text {PDEn}}$, and $c_{\text {a}}$ (cf. the values provided in the captions of Figs. 3, 4,  6 and 7). We also verified that, when the travelling wave is fully formed, the positions of the right endpoints of the supports of the cell densities are consistent with those predicted by the travelling wave analysis (i.e. those given by (51)-(53)). In more detail, denoting by $X_i$ the position of the right endpoint of the support of the cell density $n_i$ at time *t* large enough that the travelling wave is established, recalling that $z_1:= 0$ (cf. conditions (16)), we verified that the values of $Z_i:= X_i - X_1$ for $i =2, \ldots , I$ are consistent with the analytically predicted values, denoted by $Z_{\text {a}i}$, which are obtained by substituting into (51)-(53) the analytically predicted wave speed, $c_{\text {a}}$, and the numerically estimated values of $M_j$ for $j=2,\ldots ,I$.

***Impact of the parameters*** $\omega _i$ ***on the shape of travelling fronts***

The numerical results in Figs. 6 and 7 show that, in agreement with the analytical results of Theorem 1, the choice of the values of the parameters $\omega _i$ impacts on the shape of the travelling fronts that emerge. In more detail, these numerical results indicate that, once the travelling front is established, the cell densities $n_i$ are such that the interface conditions (55) are satisfied throughout the front. Hence, at the interface between the region occupied by cells with phenotype labelled by the index $i+1$ and the region occupied by cells with phenotype labelled by the index *i* (i.e. at the interface between the supports of $n_{i+1}$ and $n_{i}$), for $i=1, \ldots , I-1$: if $\omega _{i+1} > \omega _i$ then the value of the cell density at the right of the interface is smaller than the one at the left (cf. top, right panels); if $\omega _{i+1} = \omega _i$ then the value of the cell density at the right of the interface is the same as the one at the left (cf. central, right panels); if $\omega _{i+1} < \omega _i$ then the value of the cell density at the right of the interface is larger than the one at the left (cf. bottom, right panels).

## Discussion and Research Perspectives

In this work, we have considered a PDE model for the growth of heterogeneous cell populations subdivided into multiple distinct discrete phenotypes. In this model, cells preferentially move towards regions where they are less compressed, and thus their movement occurs down the gradient of the cellular pressure. The cellular pressure is defined as a weighted sum of the densities (i.e. the volume fractions) of cells with different phenotypes. To translate into mathematical terms the idea that cells with different phenotypes have different morphological and mechanical properties, both the cell mobility and the weighted amount the cells contribute to the cellular pressure vary with their phenotype. We have formally derived this model as the continuum limit of an on-lattice individual-based model, where cells are represented as single agents undergoing a branching biased random walk corresponding to phenotype-dependent and pressure-regulated cell division, death, and movement. Then, we have studied travelling wave solutions whereby cells with different phenotypes are spatially separated by sharp boundaries across the invading front (cf. Theorem 1, Remark 2, and the schematic in Fig. 2). As discussed in Batlle and Wilkinson (2012), sharp borders between distinct cell types form at the interface of both adjacent tissues and regional domains within a tissue, and the occurrence of sharp spatial segregation between cells is observed both in *Drosophila* (Irvine 2001) and in vertebrate tissues (Fraser et al. 1990; Langenberg and Brand 2005; Zeltser and Larsen 2001). Finally, we have reported on numerical simulations of the two models, demonstrating excellent agreement between them and the travelling wave analysis, thus validating the formal limiting procedure employed to derive the individual-based model from the continuum model and confirming the analytical results obtained.

The results presented here indicate that inter-cellular variability in mobility can support the maintenance of spatial segregation across invading fronts, whereby cells with a higher mobility drive invasion by occupying regions closer to the front edge. These results have been obtained under the assumption that cells with phenotypes labelled by larger values of the index *i* express a higher mobility – cf. assumptions (9) on the parameters $\gamma _i$ of the individual-based model and the corresponding assumptions (13) on the parameters $\mu _i$ of the continuum model. On the other hand, no specific assumptions have been made on the parameters $\omega _i$, which provide a measure of the weighted amount that cells with phenotype *i* contribute towards the cellular pressure (cf. the constitutive relations (3c) and (5)) and the values of which can be related, for instance, to cell stiffness – i.e. if cells with phenotype labelled by the index *i* are stiffer than cells with phenotype labelled by the index *j* then $\omega _i>\omega _j$. Therefore, the results we have presented apply both to scenarios where less stiff cells are more invasive and to opposite scenarios – scenarios that, under assumptions (9) and (13), would correspond to assuming, respectively, $\omega _{i+1}<\omega _i$ and $\omega _{i+1}>\omega _i$ for $i=1,\ldots ,I-1$. This is particularly relevant in the context of tumour growth. In fact, it has been observed, through both *in vitro* and *in vivo* experiments, that solid tumours can be made up of cells of varying stiffness (Lv et al. 2021; Swaminathan et al. 2011; Rianna et al. 2020; Baker et al. 2010; Han et al. 2020). In a large number of cancer cell lines, more migratory and invasive phenotypes align with the cells that are softest/most deformable (Lv et al. 2021; Han et al. 2020). For example, in ovarian cancer cell lines, cells with the highest migration capabilities can be up to ten times less stiff than cells with the lowest migration and invasion potential (Swaminathan et al. 2011). However, in some cell lines, such as breast cancer cell lines, stiffness has been observed to increase with tumourigenic invasive potential, especially in areas of stiff extracellular matrix (Baker et al. 2010; Mok et al. 2020).

We conclude with an outlook on possible research perspectives. In this work the focus has been placed on the *propagation* of segregation properties, and we have thus studied the existence of travelling wave solutions of the system of PDEs (3) that exhibit segregation between different cell types. Accordingly, we have carried out numerical simulations under initial conditions corresponding to scenarios where cells of different types initially occupy distinct regions of the spatial domain. As the next step, it would be relevant to investigate the *emergence* of segregation properties by studying analytically the convergence of solutions of the PDE system (3) to such travelling wave solutions, and carrying out numerical simulations in cases where cells of different types are not separated at the initial time.

It would also be interesting to explore scenarios where assumptions (7) on the parameters $\alpha _i$ are relaxed (i.e. when also proliferation and death of cells with phenotypes labelled by values of the index $i > 1$ are incorporated into the model). In this regard, under assumptions (13) on the mobility coefficients $\mu _i$, taking into account proliferation-migration trade-offs induced by the inherent energetic cost attached to cellular activities, it would be natural to consider the variant (12) of the PDE system (3) subject to assumptions (6) along with the assumptions $\alpha _1> \alpha _2> \ldots> \alpha _{I-1} > \alpha _{I} = 0$. In this case, the calculations carried out in *Step 4* of the proof of Theorem 1 would break down, hinting that conditions (20), which express the fact that the interfaces between the components $n_{i}(z)$ and $n_{i+1}(z)$ of the solution travel at the same speed *c* for all $i=1,\ldots ,I-1$, would not hold. Hence, under this scenario it is likely that the continuum model does not admit travelling wave solutions of the type of those of Theorem 1.

While, focusing our attention on spatial segregation across travelling fronts, here we have considered the case where assumptions (13) hold, this work could be extended by investigating the behaviour of solutions to the PDE system (3), and its more general variant (12), in cases where these assumptions do not hold and segregation properties may not be propagated (David et al. 2024; Lorenzi et al. 2017; Carrillo 2018a). It would also be interesting to study free boundary problems for these PDE systems, along the lines of those considered in Byrne and Chaplain (1997); Lorenzi et al. (2020); Bertsch et al. (2010) to model tissue development and tumour growth, and consider related transmission problems modelling cell invasion through thin membranes, in the vein of Chaplain (2019); Ciavolella et al. (2024); Ciavolella and Perthame (2021); Giverso et al. (2022).

Moreover, we could incorporate into the PDE system (3), or its generalised variant (12), the effects of phenotypic switching, possibly driven by the extracellular environment (Celora et al. 2021; Charras and Sahai 2014), and cell-cell adhesion, as similarly done for instance in Bubba et al. (2020); Carrillo (2018b); Crossley et al. (2024); Macfarlane et al. (2022) and Berendsen et al. (2017); Burger et al. (2020); Carrillo (2019, 2018a, 2018b), respectively. Instead of resorting to phenomenological considerations to define the additional terms modelling these phenomena, we could extend the individual-based model considered here along with the formal procedure employed to derive the corresponding continuum model so as to encompass mechanisms of phenotypic switching and cell-cell adhesion. This would be biologically interesting in that it has been shown, on the one hand, that some cancer cell lines can adaptively alter their stiffness to become softer in order to overcome physical barriers or areas of high cellular pressure (Rianna et al. 2020; Han et al. 2020), and, on the other hand, that in general internal stiffness of aggressive tumours is more heterogeneous than in quiescent tumours (Mok et al. 2020). Adapting our current model to include dynamical variation in cell stiffness through the weights modelling contribution to the cellular pressure could be of interest to study these dynamics further. On this note, recent work by Zills et al. (2023) considered a 3D individual-based model of a growing cell population where cells could become softer during cell division. The cell stiffness then related to adhesive and repulsive forces of the cells and their contribution to the local cellular pressure, which in turn regulated cell division rates. Numerical simulations of this model were able to replicate experimental data on cellular spheroids where cells towards the border were faster, larger, and softer than those at the centre of the spheroid. However, as the model considered is an individual-based model, the study in Zills et al. (2023) is based on numerical simulations only. Hence, it would be interesting to extend our modelling framework to include such additional aspects and then carry out travelling wave analysis, in order to complement numerical simulations with analytical results to facilitate a more comprehensive exploration of the model parameter space.

Another avenue for future research could be to investigate spatial segregation across travelling fronts in individual-based and continuum models for the growth of heterogeneous cell populations that encapsulate volume exclusion effects, which are not captured by the models considered here. For this, we expect modelling methods and analytical techniques similar to those employed in Baker et al. (2019); Fadai and Simpson (2020); Murphy et al. (2020, 2021); Tambyah et al. (2020) to be useful.

As a further extension of the present work, building on the modelling approach presented in David (2023); Lorenzi et al. (2023); Lorenzi and Painter (2022); Lorenzi et al. (2021), we could also let the cell phenotype vary along a spectrum, and thus be described by a continuous variable $y \in {\mathbb {R}}$ (Lorenzi et al. 2024). In this case, the evolution of the density (i.e. the volume fraction) of cells with phenotype *y* at time $t \ge 0$, *n*(*t*, *x*, *y*), would be governed by the following partial integro-differential equation model

63 $$\begin{aligned} {\left\{ \begin{array}{ll} \begin{array}{l} \displaystyle {\partial _t n - \mu (y) \, \partial _x \left( n \, \partial _x p \right) = \alpha (y) \, G(p) \, n, \quad y \in {\mathbb {R}}}, \\ \\ \displaystyle {p(t,x):= \int _{{\mathbb {R}}} \omega (y) \, n(t,x,y) \, \textrm{d}y,} \end{array} \quad (t,x) \in (0,\infty ) \times {\mathbb {R}}. \end{array}\right. } \end{aligned}$$

Note that, compared to the generalised form (12) of the PDE model (3), subject to assumptions (6), here the parameters $\mu _i$, $\alpha _i$, and $\omega _i$ have been replaced by the functions $\mu (y)$, $\alpha (y)$, and $\omega (y)$, respectively. We expect the model (63) to be derivable from an underlying individual-based model through a formal limiting procedure analogous to the one employed in Lorenzi et al. (2023); Macfarlane (2022).

The aforementioned extensions of the present work, which will bring new mathematical problems, will allow for further investigation into how phenotypic heterogeneity shapes invading fronts in growing cell populations.

## Data Availability

The code used for numerical simulations and data for this manuscript is available upon request.

## Formal Derivation of the Continuum Model from the Individual-Based Model

We detail the formal derivation of the PDE system (3) from the branching biased random walk underlying the individual-based model described in Sect. 2.

When cell dynamics are governed by the rules described in Sect. 2.1, using that $1+\tau G_i(p_j^k)_+-\tau G_i(p_j^k)_-=1+\tau G_i(p_j^k)$, the principle of mass balance gives the balance equation (10). Then, employing a formal procedure analogous to the one that we used in Chaplain et al. (2020); Macfarlane and Chaplain (2020); Macfarlane (2022), we define

$$\begin{aligned} n_i \equiv n_i(t,x):= n_{i,\ j}^{k},\quad n_i(t+\tau ,x):= n_{i,\ j}^{k+1},\quad n_i(t,x\pm \Delta _x):= n_{i,\ j\pm 1}^{k} \end{aligned}$$

and

$$\begin{aligned} p \equiv p(t,x):= p_{j}^{k}, \quad p(t,x\pm \Delta _x):= p_{j\pm 1}^{k}. \end{aligned}$$

From the constitutive relation (5) we find

A1 $$\begin{aligned} p(t,x):=\displaystyle {\sum _{i=1}^{I} \omega _i \ n_i(t,x)}, \end{aligned}$$

while from the balance equation (10) we obtain

$$\begin{aligned} &  n_i(t+\tau ,x)\\ &  \quad =n_i\left\{ \left( 1+\tau G_i(p)\right) \left[ 1-\frac{\gamma _i \left( p-p(t,x+\Delta _x)\right) _+}{2\overline{p}}-\frac{\gamma _i \left( p-p(t,x-\Delta _x)\right) _+}{2\overline{p}}\right] \right\} \nonumber \\ &  \qquad +n_i(t,x+\Delta _x)\left\{ \left( 1+\tau G_i(p(t,x+\Delta _x))\right) \left[ \frac{\gamma _i \left( p(t,x+\Delta _x)-p\right) _+}{2\overline{p}}\right] \right\} \\ &  \qquad +n_i(t,x-\Delta _x)\left\{ \left( 1+\tau G_i(p(t,x-\Delta _x))\right) \left[ \frac{\gamma _i \left( p(t,x-\Delta _x)-p\right) _+}{2\overline{p}}\right] \right\} .\nonumber \end{aligned}$$

Splitting the growth terms gives

$$\begin{aligned} n_i(t+\tau ,x)= &  n_i\left[ 1-\frac{\gamma _i \left( p-p(t,x+\Delta _x)\right) _+}{2\overline{p}}-\frac{\gamma _i \left( p-p(t,x-\Delta _x)\right) _+}{2\overline{p}}\right] \nonumber \\ &  +n_i(t,x+\Delta _x)\left[ \frac{\gamma _i \left( p(t,x+\Delta _x)-p\right) _+}{2\overline{p}}\right] \nonumber \\ &  +n_i(t,x-\Delta _x)\left[ \frac{\gamma _i \left( p(t,x-\Delta _x)-p\right) _+}{2\overline{p}}\right] \\ &  +\tau G_i(p)n_i\left[ 1\!-\!\frac{\gamma _i \left( p\!-\!p(t,x\!+\!\Delta _x)\right) _+}{2\overline{p}}\!-\!\frac{\gamma _i \left( p-p(t,x-\Delta _x)\right) _+}{2\overline{p}}\right] \nonumber \\ &  +\tau G_i(p(t,x+\Delta _x))n_i(t,x+\Delta _x)\left[ \frac{\gamma _i \left( p(t,x+\Delta _x)-p\right) _+}{2\overline{p}}\right] \nonumber \\ &  +\tau G_i(p(t,x-\Delta _x))n_i(t,x-\Delta _x)\left[ \frac{\gamma _i \left( p(t,x-\Delta _x)-p\right) _+}{2\overline{p}}\right] .\nonumber \end{aligned}$$

Now, assuming $n_i$ to be sufficiently regular, using that

$$\begin{aligned} n_i(t,x\pm \Delta _x)= n_i \pm \Delta _x \frac{\partial n_i}{\partial x}+\frac{\Delta _x^2}{2}\frac{\partial ^2 n_i}{\partial x^2 }+{\mathcal {O}}(\Delta _x^3), \end{aligned}$$

we formally obtain

$$\begin{aligned} n_i(t+\tau ,x)= &  n_i\left[ 1-\frac{\gamma _i \left( p-p(t,x+\Delta _x)\right) _+}{2\overline{p}}-\frac{\gamma _i \left( p-p(t,x-\Delta _x)\right) _+}{2\overline{p}}\right] \nonumber \\ &  +\left( n_i+\Delta _x \frac{\partial n_i}{\partial x}+\frac{\Delta _x^2}{2}\frac{\partial ^2 n_i}{\partial x^2 }\right) \left[ \frac{\gamma _i \left( p(t,x+\Delta _x)-p\right) _+}{2\overline{p}}\right] \nonumber \\ &  +\left( n_i-\Delta _x \frac{\partial n_i}{\partial x}+\frac{\Delta _x^2}{2}\frac{\partial ^2 n_i}{\partial x^2 }\right) \left[ \frac{\gamma _i \left( p(t,x-\Delta _x)-p\right) _+}{2\overline{p}}\right] \\ &  +\tau G_i(p)n_i\left[ 1\!-\!\frac{\gamma _i \left( p\!-\!p(t,x+\!\Delta _x)\right) _+}{2\overline{p}}\!-\!\frac{\gamma _i \left( p\!-\!p(t,x-\Delta _x)\right) _+}{2\overline{p}}\right] \nonumber \\ &  +\tau G_i(p(t,x+\Delta _x))\left( n_i+\Delta _x \frac{\partial n_i}{\partial x}+\frac{\Delta _x^2}{2}\frac{\partial ^2 n_i}{\partial x^2 }\right) \\ &  \left[ \frac{\gamma _i \left( p(t,x+\Delta _x)-p\right) _+}{2\overline{p}}\right] \nonumber \\ &  +\tau G_i(p(t,x-\Delta _x))\left( n_i-\Delta _x \frac{\partial n_i}{\partial x}+\frac{\Delta _x^2}{2}\frac{\partial ^2 n_i}{\partial x^2 }\right) \\ &  \left[ \frac{\gamma _i \left( p(t,x-\Delta _x)-p\right) _+}{2\overline{p}}\right] +{\mathcal {O}}(\Delta _x^3).\nonumber \end{aligned}$$

Furthermore, rearranging terms yields

$$\begin{aligned} n_i(t+\tau ,x)= &  n_i\left[ 1-\frac{\gamma _i \left( p-p(t,x+\Delta _x)\right) _+}{2\overline{p}}-\frac{\gamma _i \left( p-p(t,x-\Delta _x)\right) _+}{2\overline{p}}\right] \nonumber \\ &  +n_i\left[ \frac{\gamma _i \left( p(t,x+\Delta _x)-p\right) _+}{2\overline{p}}+\frac{\gamma _i \left( p(t,x-\Delta _x)-p\right) _+}{2\overline{p}}\right] \nonumber \\ &  +\Delta _x \frac{\partial n_i}{\partial x}\left[ \frac{\gamma _i \left( p(t,x+\Delta _x)-p\right) _+}{2\overline{p}}- \frac{\gamma _i \left( p(t,x-\Delta _x)-p\right) _+}{2\overline{p}}\right] \nonumber \\ &  +\frac{\Delta _x^2}{2}\frac{\partial ^2 n_i}{\partial x^2 }\left[ \frac{\gamma _i \left( p(t,x+\Delta _x)-p\right) _+}{2\overline{p}}+ \frac{\gamma _i \left( p(t,x-\Delta _x)-p\right) _+}{2\overline{p}}\right] \\ &  +\tau G_i(p)n_i\left[ 1\!-\!\frac{\gamma _i \left( p-p(t,x\!+\!\Delta _x)\right) _+}{2\overline{p}}\!-\!\frac{\gamma _i \left( p-p(t,x-\Delta _x)\right) _+}{2\overline{p}}\right] \nonumber \\ &  +\tau G_i(p)n_i\left[ \frac{\gamma _i \left( p(t,x+\Delta _x)-p\right) _+}{2\overline{p}}+\frac{\gamma _i \left( p(t,x-\Delta _x)-p\right) _+}{2\overline{p}}\right] \nonumber \\ &  +\Delta _x \frac{\partial n_i}{\partial x}\left[ \tau G_i(p(t,x+\Delta _x))\frac{\gamma _i \left( p(t,x+\Delta _x)-p\right) _+}{2\overline{p}}\right] \nonumber \\ &  -\Delta _x \frac{\partial n_i}{\partial x}\left[ \tau G_i(p(t,x-\Delta _x))\frac{\gamma _i \left( p(t,x-\Delta _x)-p\right) _+}{2\overline{p}}\right] \nonumber \\ &  +\frac{\Delta _x^2}{2} \frac{\partial ^2 n_i}{\partial x^2}\left[ \tau G_i(p(t,x+\Delta _x))\frac{\gamma _i \left( p(t,x+\Delta _x)-p\right) _+}{2\overline{p}}\right] \nonumber \\ &  +\frac{\Delta _x^2}{2} \frac{\partial ^2 n_i}{\partial x^2}\left[ \tau G_i(p(t,x-\Delta _x))\frac{\gamma _i \left( p(t,x-\Delta _x)-p\right) _+}{2\overline{p}}\right] +{\mathcal {O}}(\Delta _x^3).\nonumber \end{aligned}$$

Then, using the property that $(f)_+ -(-f)_+=(f)_+ -(f)_-=f$, we can simplify the latter equation to

$$\begin{aligned} n_i(t+\tau ,x)= &  n_i\left[ 1+\frac{\gamma _i \left( p(t,x+\Delta _x)-p\right) }{2\overline{p}}+\frac{\gamma _i \left( p(t,x-\Delta _x)-p\right) }{2\overline{p}}\right] \nonumber \\ &  +\Delta _x \frac{\partial n_i}{\partial x}\left[ \frac{\gamma _i \left( p(t,x+\Delta _x)-p\right) _+}{2\overline{p}}- \frac{\gamma _i \left( p(t,x-\Delta _x)-p\right) _+}{2\overline{p}}\right] \nonumber \\ &  +\frac{\Delta _x^2}{2}\frac{\partial ^2 n_i}{\partial x^2 }\left[ \frac{\gamma _i \left( p(t,x+\Delta _x)-p\right) _+}{2\overline{p}}+ \frac{\gamma _i \left( p(t,x-\Delta _x)-p\right) _+}{2\overline{p}}\right] \\ &  +\tau G_i(p)n_i\left[ 1+\frac{\gamma _i \left( p(t,x+\Delta _x)-p\right) }{2\overline{p}}+\frac{\gamma _i \left( p(t,x-\Delta _x)-p\right) }{2\overline{p}}\right] \nonumber \\ &  +\Delta _x \frac{\partial n_i}{\partial x}\left[ \tau G_i(p(t,x+\Delta _x))\frac{\gamma _i \left( p(t,x+\Delta _x)-p\right) _+}{2\overline{p}}\right] \nonumber \\ &  -\Delta _x \frac{\partial n_i}{\partial x}\left[ \tau G_i(p(t,x-\Delta _x))\frac{\gamma _i \left( p(t,x-\Delta _x)-p\right) _+}{2\overline{p}}\right] \nonumber \\ &  +\frac{\Delta _x^2}{2} \frac{\partial ^2 n_i}{\partial x^2}\left[ \tau G_i(p(t,x+\Delta _x))\frac{\gamma _i \left( p(t,x+\Delta _x)-p\right) _+}{2\overline{p}}\right] \nonumber \\ &  +\frac{\Delta _x^2}{2} \frac{\partial ^2 n_i}{\partial x^2}\left[ \tau G_i(p(t,x-\Delta _x))\frac{\gamma _i \left( p(t,x-\Delta _x)-p\right) _+}{2\overline{p}}\right] \nonumber \\ &  +{\mathcal {O}}(\Delta _x^3),\nonumber \end{aligned}$$

from which, if *p* is sufficiently regular, using that

$$\begin{aligned} p(t,x\pm \Delta _x)=p\pm \Delta _x \frac{\partial p}{\partial x}+\frac{\Delta _x^2}{2}\frac{\partial ^2 p}{\partial x^2}+{\mathcal {O}}(\Delta _x^3), \end{aligned}$$

we formally obtain

$$\begin{aligned} n_i(t+\tau ,x)= &  n_i\left[ 1+\frac{\gamma _i \left( \Delta _x \frac{\partial p}{\partial x}+\frac{\Delta _x^2}{2}\frac{\partial ^2 p}{\partial x^2}\right) }{2\overline{p}}+\frac{\gamma _i \left( -\Delta _x \frac{\partial p}{\partial x}+\frac{\Delta _x^2}{2}\frac{\partial ^2 p}{\partial x^2}\right) }{2\overline{p}}\right] \nonumber \\ &  +\Delta _x \frac{\partial n_i}{\partial x}\left[ \frac{\gamma _i \left( \Delta _x \frac{\partial p}{\partial x}+\frac{\Delta _x^2}{2}\frac{\partial ^2 p}{\partial x^2}\right) _+}{2\overline{p}}- \frac{\gamma _i \left( -\Delta _x \frac{\partial p}{\partial x}+\frac{\Delta _x^2}{2}\frac{\partial ^2 p}{\partial x^2}\right) _+}{2\overline{p}}\right] \nonumber \\ &  +\frac{\Delta _x^2}{2}\frac{\partial ^2 n_i}{\partial x^2 }\left[ \frac{\gamma _i \left( \Delta _x \frac{\partial p}{\partial x}+\frac{\Delta _x^2}{2}\frac{\partial ^2 p}{\partial x^2}\right) _+}{2\overline{p}}+ \frac{\gamma _i \left( -\Delta _x \frac{\partial p}{\partial x}+\frac{\Delta _x^2}{2}\frac{\partial ^2 p}{\partial x^2}\right) _+}{2\overline{p}}\right] \\ &  +\tau G_i(p)n_i\left[ 1+\frac{\gamma _i \left( \Delta _x \frac{\partial p}{\partial x}+\frac{\Delta _x^2}{2}\frac{\partial ^2 p}{\partial x^2}\right) }{2\overline{p}}+\frac{\gamma _i \left( -\Delta _x \frac{\partial p}{\partial x}+\frac{\Delta _x^2}{2}\frac{\partial ^2 p}{\partial x^2}\right) }{2\overline{p}}\right] \nonumber \\ &  +\Delta _x \frac{\partial n_i}{\partial x}\left[ \tau G_i\left( p+\Delta _x \frac{\partial p}{\partial x}+\frac{\Delta _x^2}{2}\frac{\partial ^2 p}{\partial x^2}\right) \frac{\gamma _i \left( \Delta _x \frac{\partial p}{\partial x}+\frac{\Delta _x^2}{2}\frac{\partial ^2 p}{\partial x^2}\right) _+}{2\overline{p}}\right] \nonumber \\ &  -\Delta _x \frac{\partial n_i}{\partial x}\left[ \tau G_i\left( p-\Delta _x \frac{\partial p}{\partial x}+\frac{\Delta _x^2}{2}\frac{\partial ^2 p}{\partial x^2}\right) \frac{\gamma _i \left( -\Delta _x \frac{\partial p}{\partial x}+\frac{\Delta _x^2}{2}\frac{\partial ^2 p}{\partial x^2}\right) _+}{2\overline{p}}\right] \nonumber \\ &  +\frac{\Delta _x^2}{2} \frac{\partial ^2 n_i}{\partial x^2}\left[ \tau G_i\left( p+\Delta _x \frac{\partial p}{\partial x}+\frac{\Delta _x^2}{2}\frac{\partial ^2 p}{\partial x^2}\right) \frac{\gamma _i \left( \Delta _x \frac{\partial p}{\partial x}+\frac{\Delta _x^2}{2}\frac{\partial ^2 p}{\partial x^2}\right) _+}{2\overline{p}}\right] \nonumber \\ &  +\frac{\Delta _x^2}{2} \frac{\partial ^2 n_i}{\partial x^2}\left[ \tau G_i\left( p-\Delta _x \frac{\partial p}{\partial x}+\frac{\Delta _x^2}{2}\frac{\partial ^2 p}{\partial x^2}\right) \frac{\gamma _i \left( -\Delta _x \frac{\partial p}{\partial x}+\frac{\Delta _x^2}{2}\frac{\partial ^2 p}{\partial x^2}\right) _+}{2\overline{p}}\right] \\ &  +{\mathcal {O}}(\Delta _x^3). \end{aligned}$$

Rearranging terms and neglecting terms of order higher than ${\mathcal {O}}(\Delta _x^3)$ we find

$$\begin{aligned} n_i(t+\tau ,x)= &  n_i\left[ 1+\frac{\gamma _i \Delta _x^2}{2\overline{p}} \frac{\partial ^2 p}{\partial x^2}\right] +\Delta _x \frac{\partial n_i}{\partial x}\left[ \frac{\gamma _i \left( \Delta _x \frac{\partial p}{\partial x}\right) _+}{2\overline{p}}- \frac{\gamma _i \left( -\Delta _x \frac{\partial p}{\partial x}\right) _+}{2\overline{p}}\right] \\ &  +\tau G_i(p)n_i\left[ 1+\frac{\gamma _i \Delta _x^2}{2\overline{p}} \frac{\partial ^2 p}{\partial x^2}\right] \nonumber \\ &  +\Delta _x \frac{\partial n_i}{\partial x}\left[ \tau G_i\left( p+\Delta _x \frac{\partial p}{\partial x}+\frac{\Delta _x^2}{2}\frac{\partial ^2 p}{\partial x^2}\right) \frac{\gamma _i \left( \Delta _x \frac{\partial p}{\partial x}\right) _+}{2\overline{p}}\right] \\ &  -\Delta _x \frac{\partial n_i}{\partial x}\left[ \tau G_i\left( p-\Delta _x \frac{\partial p}{\partial x}+\frac{\Delta _x^2}{2}\frac{\partial ^2 p}{\partial x^2}\right) \frac{\gamma _i \left( -\Delta _x \frac{\partial p}{\partial x}\right) _+}{2\overline{p}}\right] +{\mathcal {O}}(\Delta _x^3). \end{aligned}$$

Again, using that $(f)_+ -(-f)_+=(f)_+ -(f)_-=f$, we can simplify the above equation to

$$\begin{aligned} n_i(t+\tau ,x)= &  n_i\left[ 1+\frac{\gamma _i \Delta _x^2}{2\overline{p}} \frac{\partial ^2 p}{\partial x^2}\right] +\frac{\gamma _i\Delta _x^2}{2\overline{p}} \frac{\partial n_i}{\partial x}\frac{\partial p}{\partial x} +\tau G_i(p)n_i\left[ 1+\frac{\gamma _i \Delta _x^2}{2\overline{p}} \frac{\partial ^2 p}{\partial x^2}\right] \\ &  +\Delta _x \frac{\partial n_i}{\partial x}\left[ \tau G_i\left( p+\Delta _x \frac{\partial p}{\partial x}+\frac{\Delta _x^2}{2}\frac{\partial ^2 p}{\partial x^2}\right) \frac{\gamma _i \left( \Delta _x \frac{\partial p}{\partial x}\right) _+}{2\overline{p}}\right] \\ &  -\Delta _x \frac{\partial n_i}{\partial x}\left[ \tau G_i\left( p-\Delta _x \frac{\partial p}{\partial x}+\frac{\Delta _x^2}{2}\frac{\partial ^2 p}{\partial x^2}\right) \frac{\gamma _i \left( -\Delta _x \frac{\partial p}{\partial x}\right) _+}{2\overline{p}}\right] +{\mathcal {O}}(\Delta _x^3), \end{aligned}$$

from which, rearranging again terms, we obtain

$$\begin{aligned} n_i(t+\tau ,x)= &  n_i+\frac{\gamma _i \Delta _x^2}{2\overline{p}}\left[ n_i \frac{\partial ^2 p}{\partial x^2}+ \frac{\partial n_i}{\partial x}\frac{\partial p}{\partial x}\right] \\ &  +\tau G_i(p)n_i +\frac{\gamma _i \Delta _x^2 \tau }{2\overline{p}} G_i(p)n_i\frac{\partial ^2 p}{\partial x^2}\\ &  +\frac{\gamma _i \Delta _x^2 \tau }{2\overline{p}}\frac{\partial n_i}{\partial x}\left[ \alpha _iG\left( p+\Delta _x \frac{\partial p}{\partial x}+\frac{\Delta _x^2}{2}\frac{\partial ^2 p}{\partial x^2}\right) \left( \frac{\partial p}{\partial x}\right) _+\right. \\ &  \left. -G_i\left( p-\Delta _x \frac{\partial p}{\partial x}+\frac{\Delta _x^2}{2}\frac{\partial ^2 p}{\partial x^2}\right) \left( -\frac{\partial p}{\partial x}\right) _+\right] \\ &  +{\mathcal {O}}(\Delta _x^3). \end{aligned}$$

Now taking $n_i$ over to the left-hand side and dividing through by $\tau$ yields

$$\begin{aligned} \frac{n_i(t+\tau ,x)-n_i}{\tau }= &  \frac{\gamma _i \Delta _x^2}{2\tau \overline{p}}\left[ n_i \frac{\partial ^2 p}{\partial x^2}+ \frac{\partial n_i}{\partial x}\frac{\partial p}{\partial x}\right] \\ &  + G_i(p)n_i+\frac{\gamma _i \Delta _x^2 }{2\overline{p}} G_i(p)n_i\frac{\partial ^2 p}{\partial x^2}\\ &  +\frac{\gamma _i \Delta _x^2 }{2\overline{p}}\frac{\partial n_i}{\partial x}\left[ G_i\left( p+\Delta _x \frac{\partial p}{\partial x}+\frac{\Delta _x^2}{2}\frac{\partial ^2 p}{\partial x^2}\right) \left( \frac{\partial p}{\partial x}\right) _+\right. \\ &  \left. -G_i\left( p-\Delta _x \frac{\partial p}{\partial x}+\frac{\Delta _x^2}{2}\frac{\partial ^2 p}{\partial x^2}\right) \left( -\frac{\partial p}{\partial x}\right) _+\right] \\ &  +{\mathcal {O}}(\Delta _x^3). \end{aligned}$$

Finally, letting $\Delta _x\rightarrow 0$ and $\tau \rightarrow 0$ in such a way that

$$\begin{aligned} \frac{\gamma _i \Delta _x^2}{2\tau \overline{p}}\rightarrow \mu _i, \quad \text {where} \quad \mu _i \in {\mathbb {R}}^*_+,\ i=1, \ldots , I, \end{aligned}$$

we formally obtain the following PDEs

$$\begin{aligned} \frac{\partial n_i}{\partial t}= &  \mu _i\left( n_i \frac{\partial ^2 p}{\partial x^2}+ \frac{\partial n_i}{\partial x}\frac{\partial p}{\partial x}\right) + G_i(p)n_i, \quad i=1,\ldots ,I, \quad (t,x) \in (0,\infty ) \times {\mathbb {R}}, \nonumber \end{aligned}$$

subject to the assumptions (13) on the mobility coefficients, $\mu _i$, which descend from assumptions (9) when conditions (11) hold. The above PDEs alongside the constitutive relation (A1) can then be rewritten as the continuum model (12).

## Addendum to *Step 8* of the Proof of Theorem 1

Differentiating the differential equation (54a) with respect to *z* gives

$$\begin{aligned} - c \, \left( p'\right) ' - \mu _1 \left[ p \, (p')'' + 2 \, p' \, (p')' + p'' \, p' \right] = \left[ \alpha _1 \, \frac{\textrm{d}G}{\textrm{d}p} \, p + \alpha _1 \, G\left( p\right) \right] \, p', \end{aligned}$$

and, given the conditions (54b), we complement the above differential equation with the following boundary conditions

$$\begin{aligned} p'(- \infty ) = 0, \qquad p'(0^-) = -\frac{c}{\mu _1}. \end{aligned}$$

On the other hand, differentiating the differential equation (54a) with respect to *c* yields

$$\begin{aligned} &  - c \, \left( \frac{\partial p}{\partial c}\right) ' - \mu _1 \left[ p \, \left( \frac{\partial p}{\partial c}\right) '' + 2 \, p' \, \left( \frac{\partial p}{\partial c}\right) ' + p'' \, \frac{\partial p}{\partial c} \right] \\ &  \quad = \left[ \alpha _1 \, \frac{\textrm{d}G}{\textrm{d}p} \, p + \alpha _1 \, G\left( p\right) \right] \, \frac{\partial p}{\partial c} + p' \end{aligned}$$

and, given the conditions (54b), we complement the above differential equation with the following boundary conditions

$$\begin{aligned} \frac{\partial p}{\partial c}(- \infty ) = 0, \qquad \left( \frac{\partial p}{\partial c}\right) '(0^-) = -\frac{1}{\mu _1}. \end{aligned}$$

Introducing the notation $f:= p'$ and $\displaystyle {g:= \frac{\partial p}{\partial c}}$, the above differential equations along with the corresponding boundary conditions can be rewritten, respectively, as  and  where the coefficients $a_1, \ldots , a_4$ are implicitly defined. Since $f<0$ on $(-\infty ,0)$ and $g'(0^-)<0$, noting that both $f(- \infty ) = 0$ and $g(- \infty ) = 0$ and the right-hand side of the differential equation (B3a) contains the additional term $f<0$ compared to the right-hand side of the differential equation (B2a), we deduce that $\displaystyle {g = \frac{\partial p}{\partial c} < 0}$ on $(-\infty ,0)$, from which we conclude that $c \mapsto p(0^-)$ is monotonically decreasing.

## Numerical Scheme for the PDE Model (3)

To construct numerical solutions of the PDE system (3) we use a finite-volume scheme with an upwind stabilisation as well as a MUSCL reconstruction of interface values to increase the spatial order of accuracy. This scheme is built upon those we employed in Bubba et al. (2020); Lorenzi et al. (2023). The full details are provided below.

We first discretise the 1D domain into spatial cells of size $\Delta x$. The time discretisation is a forward Euler method. We allow the time-step to vary during the simulation using the CFL-like condition

$$\begin{aligned} \frac{\Delta t}{\Delta x} \max (\mu _1,\ldots ,\mu _I) \max _x( |\nabla p|) < 1. \end{aligned}$$

For each spatial cell interface, we reconstruct the values of the cell densities adopting a MUSCL approach. For the $i^{th}$ component of the solution we have

$$\begin{aligned} n^L_{i(j+1/2)}= &  n^k_{i(j)} + 0.5 \phi (r_{j})(n^k_{i(j+1)}-n^k_{i(j)}), \\ n^R_{i(j+1/2)}= &  n^k_{i(j)} - 0.5 \phi (r_{j+1})(n^k_{i(j+2)}-n^k_{i(j+1)}),\\ n^L_{i(j-1/2)}= &  n^k_{i(j-1)} + 0.5 \phi (r_{j-1})(n^k_{i(j)}-n^k_{i(j-1)}), \\ n^R_{i(j-1/2)}= &  n^k_{i(j)} - 0.5 \phi (r_{j})(n^k_{i(j+1)}-n^k_{i(j)}), \end{aligned}$$

where

$$\begin{aligned} r_j=\frac{n^k_{i(j)}-n^k_{i(j-1)}}{n^k_{i(j+1)}-n^k_{i(j)}}, \end{aligned}$$

and the function $\phi (\cdot )$ is the chosen flux-limiter. In particular, here we choose the *ospre* flux-limiter, i.e.

$$\begin{aligned} \phi (r)=\frac{1.5 \left( r^2+r\right) }{\left( r^2+r+1\right) }. \end{aligned}$$

We then compute the gradient of the pressure at each cell interface. To do so, we first define

$$\begin{aligned} p_{j+1/2}^L=\sum _{i=1}^I \omega _i n_{i(j+1/2)}^L, \quad p_{j+1/2}^R=\sum _{i=1}^I \omega _i n_{i(j+1/2)}^R. \end{aligned}$$

We then compute the quantities $\nabla p_{j+1/2}^L$ and $\nabla p_{j+1/2}^R$ using: a central differencing scheme in the interior of the spatial domain; a forward differencing scheme at the left endpoint of the spatial domain; and a backward differencing scheme at the right endpoint of the spatial domain. Finally, we employ the reconstruction

$$\begin{aligned} (\nabla p)_{j+1/2}= 0.5((\nabla p)_{j+1/2}^L+ (\nabla p)_{j+1/2}^R ). \end{aligned}$$

The flux across the spatial cell interface for the discretisation of the $i^{th}$ component of the solution is given by

$$\begin{aligned} F_{j+1/2}^{n_i} = \mu _i \, n^L_{i(j+1/2)} \max (0, -(\nabla p)_{j+1/2}) + \mu _i\, n^R_{i(j+1/2)} \min (0, -(\nabla p)_{j+1/2}). \end{aligned}$$

Altogether, upon splitting between conservative and non-conservative terms, the numerical scheme reads as

$$\begin{aligned} n^{k+1/2}_{i(j)}= &  n^k_{i(j)} + \Delta t \left[ - \frac{F^{n_i}_{j+1/2}-F^{n_i}_{j-1/2}}{\Delta x} \right] ,\\ n^{k+1}_{i(j)}= &  n^{k+1/2}_{i(j)} + \Delta t \, \alpha _i \, n^{k+1/2}_{i(j)} G(p_{j}^k). \end{aligned}$$
